# HDBR Expression: A Unique Resource for Global and Individual Gene Expression Studies during Early Human Brain Development

**DOI:** 10.3389/fnana.2016.00086

**Published:** 2016-10-26

**Authors:** Susan J. Lindsay, Yaobo Xu, Steven N. Lisgo, Lauren F. Harkin, Andrew J. Copp, Dianne Gerrelli, Gavin J. Clowry, Aysha Talbot, Michael J. Keogh, Jonathan Coxhead, Mauro Santibanez-Koref, Patrick F. Chinnery

**Affiliations:** ^1^Institute of Genetic Medicine, Newcastle UniversityNewcastle upon Tyne, UK; ^2^Institute of Neuroscience, Newcastle UniversityNewcastle upon Tyne, UK; ^3^Institute of Child Health, University College LondonLondon, UK

**Keywords:** human, embryo, fetal, RNAseq, SNP genotyping, HDBR

This paper describes a new resource, HDBR (Human Developmental Biology Resource) Expression, for studying prenatal human brain development. It is unique in the age range (4 post conception weeks [PCW] to 17PCW) and number of brains (172) studied, particularly those under 8PCW (33). The great majority of the samples are karyotyped. HDBR Expression is also unique in that both the large-scale data sets (RNA-seq data, SNP genotype data) and the corresponding RNA and DNA samples are available, the latter via the MRC-Wellcome Trust funded HDBR^1^(Gerrelli et al., 2015). There are 557 RNA-seq datasets from different brain regions, the majority between 4 and 12PCW. During this time the major brain regions are established and the early stages of cortex development occur (Bystron et al., 2008; O'Rahilly and Muller, 2008). In addition, there are 42 RNAseq data sets from spinal cord and 29 from cerebral choroid plexus. There are also 243 additional tissue specimens in paraffin wax blocks available for individual gene expression studies. For almost all of the brains and specimens in wax blocks there are corresponding SNP genotype data.

Large-scale/high-throughput studies, such as next-generation sequencing, are providing raw material in a wide variety of research fields (for review of concepts and methodologies of RNA-seq, see Shin et al., 2014). Studies of human development are hampered by difficulties in obtaining tissue which means that publicly available large-scale data sets are particularly useful because data can be used and re-used (Kang et al., 2011; Zhang et al., 2011; Fietz et al., 2012; Miller et al., 2014; Darmanis et al., 2015).

## Materials and methods

### Human tissues

Human embryonic and fetal tissues were obtained from the MRC/Wellcome-Trust funded Human Developmental Biology Resource. HDBR is a tissue bank regulated by the UK Human Tissue Authority (HTA^2^) and operating in line with the relevant HTA Codes of Practice. Tissue samples are collected with appropriate maternal written consent and approval from the NRES Committee North East - Newcastle and North Tyneside 1 (REC reference 08/H0906/21+5) or NRES Committee London-Fulham (REC reference 08/H0712/34+5).

Tissues were collected over a period of 11 years (February 2003–January 2014) with the majority (82%) collected between January 2010 and January 2014 (01/28/2014 last collection date). Tissues were either fixed at 4°C in 4% paraformaldehyde and embedded in paraffin wax following standard protocols (Bussolati et al., 2011) or dissected (as described below) and tissues frozen at −80°C for RNA and DNA preparation. For the embryos and fetuses that were fixed and embedded, a small sample of the embryonic-derived part of the placenta or skin tissue was taken for DNA preparation.

There were three sets of tissues: (a) samples of embryonic-derived placenta or skin from embryos or fetuses that had been fixed and paraffin wax embedded. These tissues were used solely for DNA preparation and SNP genotyping. (b) Brain tissues where each sample was subdivided and part used for RNA preparation and part used for DNA preparation, followed by RNA-sequencing and SNP genotyping respectively. (c) Brain tissues where each sample was subdivided and part used for RNA preparation and part used for DNA preparation, followed only by RNA-sequencing. This meant that, where tissues from several different brain regions (and/or spinal cord, and/or choroid plexus) were collected from individual human embryos or fetuses, SNP genotyping was only carried out once. However, DNA was prepared separately from each of the regions sampled for a particular individual embryo or fetus, and these are available for future studies, e.g., epigenetic analysis (Reilly et al., 2015).

### Brain tissue dissections

**Figure 1:**
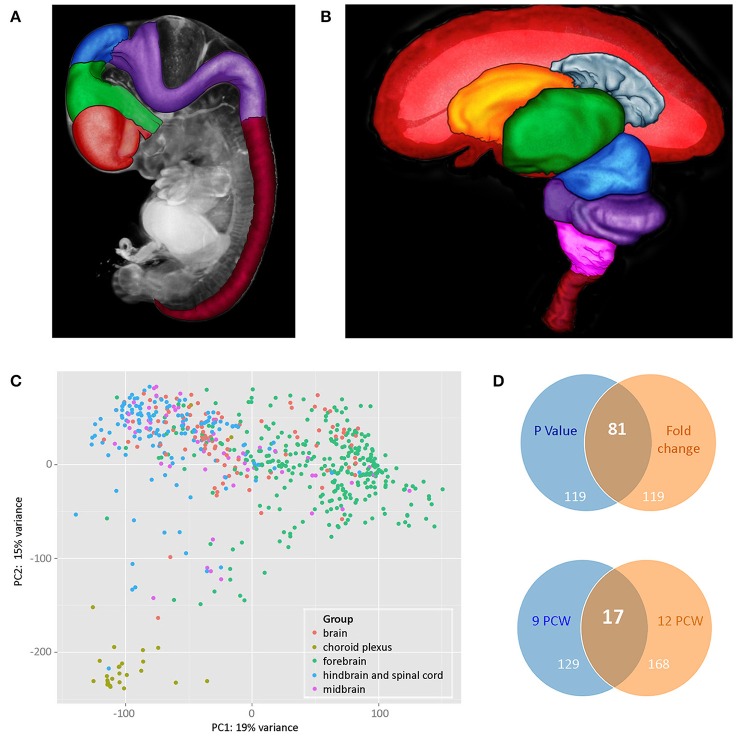
**(A,B)** Illustrate brain regions at two developmental stages: **(A)** 7 PCW. Regions of the brain plus the spinal cord have been defined in a 3 dimensional (3D) model of a Carnegie Stage 19 (CS19) embryo generated by optical projection tomography (Sharpe et al., 2002). Red—telencephalon, green—diencephalon, blue—midbrain, purple—hindbrain, deep red—spinal cord, gray—rest of head, and body. **(B)** 10 PCW. A 3D model of the brain and part of the spinal cord was generated by magnetic resonance imaging and brain regions defined. The front of the brain is on the left. In the image, the left cerebral cortex has been removed to show the inner view of the right cerebral cortex plus the inner structures of the telencephalon (choroid plexus and basal ganglia) as well as structures that lie between the two cerebral cortexes (diencephalon and midbrain) which are fully or partially hidden when looking at a whole brain of this age. Red—cerebral cortex, gray—cerebral choroid plexus, orange—basal ganglia, green—diencephalon, blue—midbrain, purple—cerebellum and pons, pink—medulla oblongata, deep red—upper part of spinal cord. For both images, the 3D models were visualized and brain regions were defined using MAPaint, custom-designed software from the Edinburgh Mouse Atlas Project team^3^. **(C)** Shows principal component analysis (PCA) analysis carried out on all RNAseq datasets. Choroid plexus samples (khaki green) provide the most distinct set. Forebrain (green) and hindbrain (blue) samples separated out but with some slight overlap. Midbrain samples (purple) and unidentified brain samples (red) fell within the forebrain and hindbrain clusters. **(D)** Shows Venn diagrams comparing genes that are differentially expressed in a subset of RNAseq data sets from cortical samples at 9 and 12 PCW. The upper panel compares the top 200 genes expressed differentially between 9 and 12 PCW (anterior, central, posterior, and temporal cortex samples grouped for each age) where the expression differences had the lowest *p*-values with the top 200 differentially expressed genes with the largest fold changes. There were 81 genes that were identified as differentially expressed between 9 and 12 PCW where the expression differences had both the lowest *p*-values and showed the highest fold change. All 200 genes with the largest fold change had a *p*-value < 0.05. The lower panel compares genes differentially expressed between anterior and posterior cortex at the two stages. At 9 PCW, 146 genes were differentially expressed between the anterior and posterior cortex. At 12 PCW, 185 genes were differentially expressed between the anterior and posterior cortex. 17 of these genes were differentially expressed between the anterior and posterior cortex at both 9 and 12 PCW. The lists of differentially expressed genes corresponding to those summarized in the upper and lower panels are shown in Supplementary Tables 3, 4, respectively, on the HDBR website.

The dissection protocol depended on the developmental stage of the embryo or fetus, reflecting the size of the brain, and the state of disruption of the tissue. The aim was to dissect brains into forebrain, midbrain and hindbrain and at later stages, to further dissect the forebrain into: (1) telencephalon (left and right in some cases) and diencephalon or (2) cortex (left and right in some cases; temporal lobe removed and it and the remaining cortex divided into strips depending on size), basal ganglia and diencephalon; hindbrain (cerebellum and medulla). The midbrain was collected as a single sample except in a few cases where it was dissected into left and right parts. Figures 1A,B show brains at two developmental stages (7PCW and 10PCW, respectively) highlighting the areas that were dissected. Where the cortex was divided into strips, this was done evenly across the cortex. In most cases five strips were generated but in some cases this varied because of the size of the brain (Ip et al., 2010). In all cases the most anterior strip was labeled 1 and the strips numbered sequentially from there toward the most posterior strip (usually labeled 5).

We also collected 29 cerebral choroid plexus samples and 42 spinal cord samples. There are also 99 samples where the region of brain could not be determined and these are simply labeled “brain fragments.”

The tissues were sent to AROS Applied Biotechnology^4^ who prepared DNA and RNA and carried out SNP genotyping and RNA-sequencing as described below.

### DNA and RNA preparation

DNA was extracted from 435 human embryonic and fetal tissue samples on the QIAsymphony SP using manufacturer's^5^ protocol DNA HC. DNA was quantified on the QuBit system (specific for dsDNA).

RNA was extracted from 705 human embryonic and fetal tissue samples. After lysis using the TissueLyser and removal of fat from the sample with chloroform, RNA (including small RNAs) was purified on the QIAsymphony SP using protocol miRNA v05. The RNA yield was estimated using Nanodrop A260 measurement and the quality evaluated for approximately 15% of the samples using an Agilent Bioanalyzer. Seventy samples had either too little RNA or the RNA was of insufficient quality. A further 3 samples failed the quality control tests at the library preparation stage (see below), 4 samples were excluded because they did not match their corresponding DNA genotyping data meaning that RNAseq datasets were obtained from 628 tissue samples in total.

### SNP genotyping

SNP genotyping was carried out according to the Illumina Infinium LCG Quad Assay protocol^6^. Briefly, DNA was denatured, amplified and then hybridized to Illumina's HumanOmni5-Quad BeadChip (HumanOmni5-4v1_B). Array-based single base primer extension was performed using labeled nucleotides (C and G nucleotide were biotin-labeled while A and T were dinitrophenyl-labeled). Then, after washing and drying, the BeadChips were imaged using the Illumina iScan system. After scanning the idat files were imported into the Illumina GenomeStudio software for genotyping calls and gender calls (average call rate 98.3%).

### RNA-sequencing and analysis

cDNA was generated from the RNAs using Illumina's Stranded mRNA Sample Prep Kit followed by library preparation following Illumina's guidelines for the TruSeq Stranded mRNA LT sample prep kit. Four hundred ng of total RNA was used as the input for each sample. The concentration of each library was determined using the KAPA qPCR kit (KK4835) and triplicate reactions using three independent 10^6^-fold dilutions of the libraries. The size profile of approximately 15% of the libraries was evaluated using an Agilent Bioanalyzer DNA 1000 chip. The average final library size was between 272 and 467 bp (includes 120 nucleotides of adapter sequence). The libraries were sequenced on an Illumina HiSeq2000.

RNA-seq data were processed and analyzed to identify differentially expressed genes. The quality of sequencing reads was first checked with FastQC^7^. Poly-N tails were trimmed off from reads with an in house Perl script. The 12 bp on the left ends and 4 bp on the right ends of all reads were clipped off with Seqtk^8^ to remove biased sequencing bases observed in FastQC reports. Low quality bases (*Q* < 30) and standard Illumina (Illumina, Inc. California, U.S.) paired-end sequencing adaptors on 3′ ends of reads were trimmed off using autoadapt^9^ and only those that were at least 20 bp in length after trimming were kept. The high quality reads were then mapped to the human reference genome hg38 with Tophat2 (Kim et al., 2013). Reads aligned to genes were counted with HTSeq-count (Anders et al., 2015). Differentially expressed genes were then identified with Bioconductor (Gentleman et al., 2004) package DESeq2 (Love et al., 2014).

## HDBR expression resource

Table 1 summarizes the developmental stages and tissue regions for which there is RNA-seq data. For each individual tissue sample there is information on the name of the sample (e.g., HDBR251), the ID number of the embryo or fetus which it came from (e.g., 1406), the developmental stage and karyotype, all of which can be found in the sample attributes and variables accompanying the data sets uploaded to ArrayExpress^10^ (Kolesnikov et al., 2015). For most of the samples there is also information on the time to collection and this, with the information on each sample, can also be found in Supplementary Table 1 on the HDBR website^11^. The ID number of the embryo or fetus enables all the RNA-seq data sets from a single embryo or fetus to be identified. Similarly this ID number links to the corresponding SNP genotyping data. There are also ID numbers for embryos and fetuses for which there is SNP genotyping data and a corresponding wax block available for individual gene expression analyses. Each data entry in the SNP genotyping data repository also has the information for the corresponding tissue sample from which DNA was prepared (placenta or skin) as well as what tissues are in the wax block. The information on all the SNP genotype data can also be found in Supplementary Table 2 on the HDBR website.

**Table 1:** **Developmental stage and tissue distribution of RNAseq datasets**.

**Stage (post conception week)**	**4**	**5**	**6**	**7**	**8**	**9**	**10**	**11**	**12**	**13**	**14**	**15**	**16**	**17**	**19**	**20**	**Any stage**
Brain	2	8	6	26	171	81	27	62	77	31	19	12	22	10	2	1	557
Forebrain	1	2	1	1	74	57	17	46	52	14	8	10	16	6			305
Telencephalon					48	46	11	38	45	10	7	10	15	6			236
*Cortex*					16	28	4	18	25	6	4	8	10	3			*122*
*Temporal lobe*					1	11	2	8	10	1	2	2	3	2			*42*
*Basal ganglia*					15	5	1	5	2	3			1	1			*33*
*Whole telencephalon*					*16*	*2*		*1*			*1*		*1*				*21*
*Telencephalon fragments*							*4*	*6*	*8*								*18*
Diencephalon					19	6	4	5	3	3	1		1				*42*
Whole forebrain	*1*	*2*	*1*	*1*	*3*	*3*	*2*	*2*	*3*								*18*
Forebrain fragments					*4*	*2*		*1*	*1*	*1*							*9*
Midbrain	1	2	1	1	22	8	3	4	10	3			1				*56*
Hindbrain		2	1	3	42	15	5	8	11	4	2		3	1			97
*Cerebellum*					17	5	2	4	4	2			1	1			*36*
*Pons*						1							1				*2*
*Medulla oblongata*					14	4	2	2	0	2			1				*25*
*Whole hindbrain*		*2*	*1*	*3*	*6*	*5*	*1*	*2*	*3*		*2*						*29*
*Hindbrain fragments*					*5*				*4*								*5*
Brain fragments		*2*	*3*	*21*	*33*	*1*	*2*	*4*	*4*	*10*	*9*	*2*	*2*	*3*	*2*	*1*	*99*
Spinal cord		1	2	8	16	4	4	3	3	1							42
Choroid plexus					11	6	2	3	4	1	1		1				29
																	
All	2	9	8	34	198	91	33	68	84	33	20	12	23	10	2	1	628

Each cell represents the number of RNAseq datasets for a particular tissue at each developmental stage. The totals given in the rows with the tissue labeled in red are the summation of the datasets for the tissues in indented rows below e.g., “Brain” is the sum of the Forebrain, Midbrain, Hindbrain, and “Brain fragment” cells in the column for each stage. “Fragments” is used for samples where the precise region is unknown. For ease of viewing the table the subdivisions of Telencephalon and Hindbrain (3rd level of indent) are given in italics.

The RNAseq data files are all fastq format and the raw data files for the SNP genotype have been uploaded. Both the RNAseq and SNP genotyping files are identified by the sample name which links to the sample information in the “sample attributes and variables” tab in ArrayExpress and in the Supplementary Tables on the HDBR website. The experiment number for the RNAseq data set is E-MTAB-4840 and for the SNP dataset is E-MTAB-4843. The RNAseq data set will be incorporated into the European Bioinformatics Institute (EBI) Expression Atlas^12^ which is EBI's value-added database for high-quality data from large microarray and RNA-sequencing experiments. In the latest version, Expression Atlas analyses selected large RNA sequencing experiments to produce “baseline expression,” the abundance of each gene and splice site variant from the individual biological components (e.g., tissues or cells) used in the experiment (Petryszak et al., 2014). The HDBR RNAseq dataset will provide baseline expression from different brain regions across a substantial time period of early human development (4 to 17 PCW).

### Overview of RNA-seq datasets and preliminary characterization of datasets from a subset of cortical samples

Principal component analysis (PCA) analysis was carried out based on the normalized gene expressions from the RNA-seq datasets with the samples categorized according to gross region (forebrain, midbrain, hindbrain, and spinal cord). The datasets from brain fragments and choroid plexus were also included. From Figure 1C it can be seen that there is clustering according to brain region and choroid plexus samples appear as a separate tight group.

A subset of 64 RNA-seq datasets from anterior, central, posterior, and temporal cortex taken at either 9 or 12 PCW were selected for further differential expression analysis. Figure 1D shows that there is a larger number of genes differentially expressed with age rather than cortical spatial location at these stages. It is also clear, however, that there is differential expression between anterior and posterior cortex at both stages and the evidence suggests that the expression profiles of both the anterior and posterior cortex change from 9 to 12 PCW.

## Author contributions

All authors contributed to revising the work, had final approval of the version to be published and agree to be accountable in relation to the accuracy and integrity of the work. SJL drafted the paper and PC, SJL, MS, and AC made substantial contributions to the conception and design of the work; YX, LH, GC, and MK made substantial contributions to the analysis and interpretation of data and SNL, DG, AT, and JC made substantial contributions to the acquisition of data.

## Funding

We gratefully acknowledge funding for this work from the UK Medical Research Council (grant number MC_PC_13047). PC is a Wellcome Trust Senior Fellow in Clinical Science (101876/Z/13/Z), and a UK NIHR Senior Investigator, who receives support from the Medical Research Council Mitochondrial Biology Unit (MC_UP_1501/2).

### Conflict of interest statement

The authors declare that the research was conducted in the absence of any commercial or financial relationships that could be construed as a potential conflict of interest.

## Footnotes

^1^www.hdbr.org.

^2^www.hta.gov.uk.

^3^http://www.emouseatlas.org/emap/analysis_tools_resources/software/eMAP-apps.html.

^4^http://arosab.com.

^5^https://www.qiagen.com/gb/.

^6^http://support.illumina.com/content/dam/illumina-support/documents/documentation/chemistry_documentation/infinium_assays/infinium_lcg_quad_assay/infinium-lcg-quad-assay-guide-15025908-d.pdf.

^7^http://www.bioinformatics.babraham.ac.uk/projects/fastqc/.

^8^https://github.com/lh3/seqtk.

^9^https://github.com/optimuscoprime/autoadapt.

^10^https://www.ebi.ac.uk/arrayexpress/.

^11^http://www.hdbr.org.

^12^http://www.ebi.ac.uk/gxa/.

## References

[B1] Anders S. Pyl P. T. Huber W. (2015). HTSeq–a Python framework to work with high-throughput sequencing data. Bioinformatics 31, 166–169. 10.1093/bioinformatics/btu638 25260700 PMC4287950

[B2] Bussolati G. Annaratone L. Medico E. D'Armento G. Sapino A. (2011). Formalin fixation at low temperature better preserves nucleic acid integrity. PLoS ONE 6:e21043. 10.1371/journal.pone.0021043 21698245 PMC3115967

[B3] Bystron I. Blakemore C. Rakic P. (2008). Development of the human cerebral cortex: boulder committee revisited. Nat. Rev. Neurosci. 9, 110–122. 10.1038/nrn2252 18209730

[B4] Darmanis S. Sloan S. A. Zhang Y. Enge M. Caneda C. Shuer L. M.. (2015). A survey of human brain transcriptome diversity at the single cell level. Proc. Natl. Acad. Sci. U.S.A. 112, 7285–7290. 10.1073/pnas.1507125112 26060301 PMC4466750

[B5] Fietz S. A. Lachmann R. Brandl H. Kircher M. Samusik N. Schröder R.. (2012). Transcriptomes of germinal zones of human and mouse fetal neocortex suggest a role of extracellular matrix in progenitor self-renewal. Proc. Natl. Acad. Sci. U.S.A. 109, 11836–11841. 10.1073/pnas.1209647109 22753484 PMC3406833

[B6] Gentleman R. C. Carey V. J. Bates D. M. Bolstad B. Dettling M. Dudoit S.. (2004). Bioconductor: open software development for computational biology and bioinformatics. Genome Biol. 5:R80. 10.1186/gb-2004-5-10-r80 15461798 PMC545600

[B7] Gerrelli D. Lisgo S. Copp A. J. Lindsay S. (2015). Enabling research with human embryonic and fetal tissue resources. Development 142, 3073–3076. 10.1242/dev.122820 26395135 PMC4640175

[B8] Ip B. K. Wappler I. Peters H. Lindsay S. Clowry G. J. Bayatti N. (2010). Investigating gradients of gene expression involved in early human cortical development. J. Anat. 217, 300–311. 10.1111/j.1469-7580.2010.01259.x 20579172 PMC2992409

[B9] Kang H. J. Kawasawa Y. I. Cheng F. Zhu Y. Xu X. Li M.. (2011). Spatio-temporal transcriptome of the human brain. Nature 478, 483–489. 10.1038/nature10523 22031440 PMC3566780

[B10] Kim D. Pertea G. Trapnell C. Pimentel H. Kelley R. Salzberg S. L. (2013). TopHat2: accurate alignment of transcriptomes in the presence of insertions, deletions and gene fusions. Genome Biol. 14:R36. 10.1186/gb-2013-14-4-r36 23618408 PMC4053844

[B11] Kolesnikov N. Hastings E. Keays M. Melnichuk O. Tang Y. A. Williams E.. (2015). ArrayExpress update–simplifying data submissions. Nucleic Acids Res. 43, D1113–D1116. 10.1093/nar/gku1057 25361974 PMC4383899

[B12] Love M. I. Huber W. Anders S. (2014). Moderated estimation of fold change and dispersion for RNA-seq data with DESeq2. Genome Biol. 15, 550. 10.1186/s13059-014-0550-8 25516281 PMC4302049

[B13] Miller J. A. Ding S. L. Sunkin S. M. Smith K. A. Ng L. Szafer A.. (2014). Transcriptional landscape of the prenatal human brain. Nature 508, 199–206. 10.1038/nature13185 24695229 PMC4105188

[B14] O'Rahilly R. Muller F. (2008). Significant features in the early prenatal development of the human brain. Ann. Anat. 190, 105–118. 10.1016/j.aanat.2008.01.001 18356030

[B15] Petryszak R. Burdett T. Fiorelli B. Fonseca N. A. Gonzalez-Porta M. Hastings E.. (2014). Expression Atlas update–a database of gene and transcript expression from microarray- and sequencing-based functional genomics experiments. Nucleic Acids Res. 42, D926–D932. 10.1093/nar/gkt1270 24304889 PMC3964963

[B16] Reilly S. K. Yin J. Ayoub A. E. Emera D. Leng J. Cotney J.. (2015). Evolutionary genomics. Evolutionary changes in promoter and enhancer activity during human corticogenesis. Science 347, 1155–1159. 10.1126/science.1260943 25745175 PMC4426903

[B17] Sharpe J. Ahlgren U. Perry P. Hill B. Ross A. Hecksher-Sorensen J.. (2002). Optical projection tomography as a tool for 3D microscopy and gene expression studies. Science 296, 541–545. 10.1126/science.1068206 11964482

[B18] Shin J. Ming G. L. Song H. (2014). Decoding neural transcriptomes and epigenomes via high-throughput sequencing. Nat. Neurosci. 17, 1463–1475. 10.1038/nn.3814 25349913 PMC6195198

[B19] Zhang Y. E. Landback P. Vibranovski M. D. Long M. (2011). Accelerated recruitment of new brain development genes into the human genome. PLoS Biol. 9:e1001179. 10.1371/journal.pbio.1001179 22028629 PMC3196496

