# Supplementary material for: HDBR Expression: A Unique Resource for Global and Individual Gene Expression Studies during Early Human Brain Development
Source: Front Neuroanat. 2016 Oct 26;10:86. doi: 10.3389/fnana.2016.00086 (PMC5080337; doi:10.3389/fnana.2016.00086)
Supplement: Supplementary file 1 [file Table1.pdf]

Supplementary Table 1 Details of tissues used for RNAseq

(pink)	RNAseq data from these samples, RNA and DNA available
(blue)	RNAseq and SNP genotype data from these samples, RNA and DNA available
(yellow)	RNAseq data from these samples, RNA only available

HDBR embryo or fetus #	Developmental Stage of embryo or fetus	Tissue Sample ID number	tissue morphology of sample	left or right	Karyotype	time sample in transit (minutes)
1406	CS 23	HDBR251	telencephalon	left	46, XX	240
1406	CS 23	HDBR252	midbrain		46, XX	240
1406	CS 23	HDBR253	telencephalon	right	46, XX	240
1406	CS 23	HDBR254	diencephalon		46, XX	240
11823	CS 23	HDBR255	midbrain		46, XY	120
11823	CS 23	HDBR256	telencephalon	right	46, XY	120
11823	CS 23	HDBR257	medulla oblongata		46, XY	120
11823	CS 23	HDBR258	choroid plexus	left	46, XY	120
11823	CS 23	HDBR259	telencephalon	left	46, XY	120
11823	CS 23	HDBR260	brain fragment	left	46, XY	120
11823	CS 23	HDBR261	choroid plexus	right	46, XY	120
11823	CS 23	HDBR262	brain fragment	right	46, XY	120
11823	CS 23	HDBR263	diencephalon	left	46, XY	120
11823	CS 23	HDBR264	diencephalon	right	46, XY	120
11826	CS 23	HDBR265	cortex	left	46, XX	120
11826	CS 23	HDBR266	midbrain		46, XX	120
11826	CS 23	HDBR267	medulla oblongata		46, XX	120
11826	CS 23	HDBR268	diencephalon	left	46, XX	120
11826	CS 23	HDBR269	diencephalon	right	46, XX	120
11826	CS 23	HDBR270	choroid plexus		46, XX	120
11826	CS 23	HDBR272	basal ganglia	right	46, XX	120
11826	CS 23	HDBR273	cortex	right	46, XX	120
11826	CS 23	HDBR274	brain fragment		46, XX	120
11827	CS 23	HDBR275	cortex	right	46, XX	120
11827	CS 23	HDBR276	midbrain		46, XX	120
11827	CS 23	HDBR277	medulla oblongata		46, XX	120
11827	CS 23	HDBR278	diencephalon	left	46, XX	120
11827	CS 23	HDBR279	diencephalon	right	46, XX	120
11827	CS 23	HDBR280	choroid plexus	right	46, XX	120
11827	CS 23	HDBR281	basal ganglia	left	46, XX	120
11827	CS 23	HDBR282	basal ganglia	right	46, XX	120

11827	CS 23	HDBR283	cortex	left	46, XX	120
11827	CS 23	HDBR284	brain fragment		46, XX	120
11846	CS 23	HDBR285	cortex	left	46, XX	1800
11846	CS 23	HDBR286	midbrain		46, XX	1800
11846	CS 23	HDBR287	medulla oblongata		46, XX	1800
11846	CS 23	HDBR288	cortex	right	46, XX	1800
11846	CS 23	HDBR289	diencephalon	left	46, XX	1800
11846	CS 23	HDBR290	diencephalon	right	46, XX	1800
11846	CS 23	HDBR291	basal ganglia	left	46, XX	1800
11846	CS 23	HDBR292	basal ganglia	right	46, XX	1800
11846	CS 23	HDBR293	choroid plexus	left	46, XX	1800
11846	CS 23	HDBR294	choroid plexus	right	46, XX	1800
11848	CS 23	HDBR295	forebrain	right	46, XY	120
11848	CS 23	HDBR296	hindbrain	left	46, XY	120
11848	CS 23	HDBR297	midbrain	right	46, XY	120
11848	CS 23	HDBR298	forebrain	left	46, XY	120
11848	CS 23	HDBR299	midbrain	left	46, XY	120
11884	CS 23	HDBR300	medulla oblongata		46, XX	230
11884	CS 23	HDBR301	telencephalon	left	46, XX	230
11884	CS 23	HDBR302	midbrain		46, XX	230
11884	CS 23	HDBR303	telencephalon	right	46, XX	230
11884	CS 23	HDBR304	diencephalon		46, XX	230
11918	CS 23	HDBR305	medulla oblongata		46, XX	120
11918	CS 23	HDBR306	midbrain		46, XX	120
11918	CS 23	HDBR307	choroid plexus	left	46, XX	120
11918	CS 23	HDBR308	cortex	left	46, XX	120
11918	CS 23	HDBR309	basal ganglia	left	46, XX	120
11918	CS 23	HDBR310	basal ganglia	right	46, XX	120
11918	CS 23	HDBR311	cortex	right	46, XX	120
11918	CS 23	HDBR312	choroid plexus	right	46, XX	120
11918	CS 23	HDBR313	diencephalon		46, XX	120
11860	CS 23	HDBR314	brain fragment		46, XX	120
11843	CS 23	HDBR315	brain fragment		46, XX	120
11824	CS 23	HDBR316	hindbrain		46, XY	120
11444	CS 23	HDBR317	brain fragment		46, XY	120
11814	CS 22	HDBR319	telencephalon	right	46, XY	UNKNOWN
11814	CS 22	HDBR320	midbrain	right	46, XY	UNKNOWN
11814	CS 22	HDBR321	hindbrain without cerebellum	right	46, XY	UNKNOWN
11814	CS 22	HDBR323	diencephalon and pituitary	right	46, XY	UNKNOWN
11814	CS 22	HDBR324	spinal cord	right	46, XY	UNKNOWN
11815	CS 22	HDBR325	forebrain		46, XX	UNKNOWN

11815	CS 22	HDBR326	midbrain		46, XX	UNKNOWN
11815	CS 22	HDBR327	hindbrain without cerebellum		46, XX	UNKNOWN
11820	9 pcw	HDBR328	cortex slice 1 of 4	right	46, XX	UNKNOWN
11820	9 pcw	HDBR329	temporal lobe slice 1 of 2	right	46, XX	UNKNOWN
11820	9 pcw	HDBR330	hindbrain	right	46, XX	UNKNOWN
11820	9 pcw	HDBR331	choroid plexus	left	46, XX	UNKNOWN
11820	9 pcw	HDBR332	cortex slice 1 of 4	left	46, XX	UNKNOWN
11820	9 pcw	HDBR333	cortex slice 4 of 4	right	46, XX	UNKNOWN
11820	9 pcw	HDBR334	choroid plexus	right	46, XX	UNKNOWN
11820	9 pcw	HDBR335	cortex slice 3 of 4	left	46, XX	UNKNOWN
11820	9 pcw	HDBR336	cortex slice 4 of 4	left	46, XX	UNKNOWN
11820	9 pcw	HDBR337	temporal lobe	left	46, XX	UNKNOWN
11820	9 pcw	HDBR338	midbrain	right	46, XX	UNKNOWN
11820	9 pcw	HDBR339	diencephalon		46, XX	UNKNOWN
11828	CS 22	HDBR340	medulla oblongata		46, XX	UNKNOWN
11828	CS 22	HDBR341	telencephalon	left	46, XX	UNKNOWN
11828	CS 22	HDBR342	midbrain		46, XX	UNKNOWN
11828	CS 22	HDBR343	telencephalon	right	46, XX	UNKNOWN
11829	CS 22	HDBR344	midbrain		46, XY	180
11829	CS 22	HDBR345	telencephalon	right	46, XY	180
11829	CS 22	HDBR346	medulla oblongata		46, XY	180
11829	CS 22	HDBR347	telencephalon	left	46, XY	180
11831	CS 22	HDBR348	cortex	left	46, XY	UNKNOWN
11831	CS 22	HDBR349	medulla oblongata		46, XY	UNKNOWN
11831	CS 22	HDBR350	midbrain		46, XY	UNKNOWN
11831	CS 22	HDBR351	diencephalon	left	46, XY	UNKNOWN
11831	CS 22	HDBR352	cortex	right	46, XY	UNKNOWN
11831	CS 22	HDBR353	basal ganglia	left	46, XY	UNKNOWN
11831	CS 22	HDBR354	choroid plexus	left	46, XY	UNKNOWN
11831	CS 22	HDBR355	choroid plexus	right	46, XY	UNKNOWN
11831	CS 22	HDBR356	diencephalon	right	46, XY	UNKNOWN
11831	CS 22	HDBR357	basal ganglia	right	46, XY	UNKNOWN
11858	CS 22	HDBR358	cortex		46, XY	120
11858	CS 22	HDBR359	midbrain		46, XY	120
11858	CS 22	HDBR360	medulla oblongata		46, XY	120
11865	CS 22	HDBR361	midbrain		46, XY	UNKNOWN
11865	CS 22	HDBR362	telencephalon	right	46, XY	UNKNOWN
11871	CS 22	HDBR364	telencephalon	left	46, XX	240
11871	CS 22	HDBR365	telencephalon	right	46, XX	240

11871	CS 22	HDBR366	forebrain fragment	left	46, XX	240
11871	CS 22	HDBR367	choroid plexus		46, XX	240
11871	CS 22	HDBR368	medulla oblongata		46, XX	240
11878	CS 22	HDBR369	telencephalon	right	46, XX	UNKNOWN
11878	CS 22	HDBR370	midbrain		46, XX	UNKNOWN
11878	CS 22	HDBR371	medulla oblongata	right	46, XX	UNKNOWN
11878	CS 22	HDBR372	diencephalon		46, XX	UNKNOWN
11878	CS 22	HDBR373	telencephalon	left	46, XX	UNKNOWN
11868	CS 22	HDBR374	brain fragment		46, XX	120
11793	CS 22	HDBR375	brain fragment		46, XY	120
11443	CS 22	HDBR376	brain fragment		46, XX	120
11410	CS 22	HDBR377	brain fragment		46, XY	120
11882	CS 21	HDBR378	forebrain- frontal		46, XX	120
11882	CS 21	HDBR379	hindbrain		46, XX	120
11849	CS 21	HDBR380	brain fragment		46, XY	120
11811	CS 21	HDBR381	brain fragment		46, XX	120
11856	CS 19	HDBR382	hindbrain		46, XY	120
11566	CS 19	HDBR383	brain fragment		46, XX	120
11514	CS 19	HDBR384	brain fragment		46, XX	120
11904	CS 18	HDBR387	forebrain		46, XY	UNKNOWN
11904	CS 18	HDBR388	midbrain		46, XY	UNKNOWN
11770	CS 18	HDBR389	brain fragment		46, XX	120
11627	CS 18	HDBR390	brain fragment		46, XX	120
11456	CS 18	HDBR391	brain fragment		46, XX	120
11931	CS 17	HDBR392	forebrain		UNKNOWN	UNKNOWN
11931	CS 17	HDBR393	midbrain		UNKNOWN	UNKNOWN
11931	CS 17	HDBR394	hindbrain		UNKNOWN	UNKNOWN
11947	16 pcw	HDBR395	telencephalon	right	46, XX	500
11840	CS 14	HDBR396	forebrain		46, XX	120
11840	CS 14	HDBR397	midbrain		46, XX	120
11840	CS 14	HDBR398	spinal cord		46, XX	120
11837	CS 14	HDBR399	forebrain		46, XY	180
11837	CS 14	HDBR400	midbrain		46, XY	180
11903	CS 13	HDBR401	forebrain		46, XX	315
11810	9 pcw	HDBR402	forebrain		46, XX	120
11810	9 pcw	HDBR403	midbrain		46, XX	120
11810	9 pcw	HDBR404	spinal cord		46, XX	120
11845	9 pcw	HDBR405	cortex slice 1 of 5	left	46, XY	120
11845	9 pcw	HDBR406	cortex slice 5 of 5	left	46, XY	120
11845	9 pcw	HDBR407	temporal lobe slice 1 of 2	left	46, XY	120
11845	9 pcw	HDBR408	medulla oblongata		46, XY	120

11845	9 pcw	HDBR409	midbrain		46, XY	120
11845	9 pcw	HDBR410	diencephalon	left	46, XY	120
11845	9 pcw	HDBR411	temporal lobe slice 2 of 2	left	46, XY	120
11845	9 pcw	HDBR412	diencephalon	left	46, XY	120
11845	9 pcw	HDBR413	choroid plexus	left	46, XY	120
11845	9 pcw	HDBR414	cortex slice 3 of 5	left	46, XY	120
11845	9 pcw	HDBR415	cortex slice 4 of 5	left	46, XY	120
11845	9 pcw	HDBR416	diencephalon	right	46, XY	120
11845	9 pcw	HDBR417	temporal lobe slice 1 of 2	right	46, XY	120
11845	9 pcw	HDBR418	temporal lobe slice 2 of 2	right	46, XY	120
11845	9 pcw	HDBR419	basal ganglia	right	46, XY	120
11845	9 pcw	HDBR420	choroid plexus	right	46, XY	120
11845	9 pcw	HDBR421	cortex slice 1 of 5	right	46, XY	120
11845	9 pcw	HDBR422	cortex slice 5 of 5	right	46, XY	120
11851	9 pcw	HDBR423	cortex	right	46, XX	240
11851	9 pcw	HDBR424	cortex	right	46, XX	240
11851	9 pcw	HDBR425	temporal lobe slice 1 of 2	right	46, XX	240
11851	9 pcw	HDBR426	medulla oblongata		46, XX	240
11851	9 pcw	HDBR427	midbrain		46, XX	240
11851	9 pcw	HDBR431	temporal lobe slice 2 of 2	left	46, XX	240
11851	9 pcw	HDBR432	choroid plexus		46, XX	240
11851	9 pcw	HDBR433	basal ganglia	left	46, XX	240
11851	9 pcw	HDBR434	cortex	left	46, XX	240
11851	9 pcw	HDBR435	cortex	left	46, XX	240
11851	9 pcw	HDBR436	temporal lobe slice 2 of 2	right	46, XX	240
11851	9 pcw	HDBR437	basal ganglia	right	46, XX	240
11851	9 pcw	HDBR438	cortex	right	46, XX	240
11851	9 pcw	HDBR439	cortex	right	46, XX	240
11851	9 pcw	HDBR440	cortex	right	46, XX	240
11873	9 pcw	HDBR441	cortex slice 1 of 5	right	46, XX	1080
11873	9 pcw	HDBR442	cortex slice 5 of 5	right	46, XX	1080
11873	9 pcw	HDBR443	temporal lobe slice 1 of 2	right	46, XX	1080
11873	9 pcw	HDBR444	medulla oblongata		46, XX	1080
11873	9 pcw	HDBR445	pons		46, XX	1080
11873	9 pcw	HDBR446	midbrain		46, XX	1080
11873	9 pcw	HDBR447	diencephalon	left	46, XX	1080
11873	9 pcw	HDBR449	basal ganglia	left	46, XX	1080

11873	9 pcw	HDBR450	cortex slice 1 of 5	left	46, XX	1080
11873	9 pcw	HDBR451	cortex slice 3 of 5	left	46, XX	1080
11873	9 pcw	HDBR452	cortex slice 4 of 5	left	46, XX	1080
11873	9 pcw	HDBR453	cortex slice 5 of 5	left	46, XX	1080
11873	9 pcw	HDBR454	temporal lobe slice 2 of 2	right	46, XX	1080
11873	9 pcw	HDBR455	diencephalon	right	46, XX	1080
11873	9 pcw	HDBR456	choroid plexus	right	46, XX	1080
11873	9 pcw	HDBR457	basal ganglia	right	46, XX	1080
11606	9 pcw	HDBR458	forebrain	left	46, XY	UNKNOWN
11606	9 pcw	HDBR459	forebrain	right	46, XY	UNKNOWN
11606	9 pcw	HDBR460	midbrain		46, XY	UNKNOWN
11912	9 pcw	HDBR461	telencephalon	right	46, XY	120
11912	9 pcw	HDBR462	telencephalon	left	46, XY	120
11912	9 pcw	HDBR463	midbrain		46, XY	120
11912	9 pcw	HDBR464	medulla oblongata		46, XY	120
11874	9 pcw	HDBR465	forebrain fragment		46, XY	120
11874	9 pcw	HDBR466	midbrain		46, XY	120
11881	9 pcw	HDBR468	hindbrain		46, XY	120
11626	9 pcw	HDBR469	forebrain fragment		46, XX	120
11832	Late 8 pcw	HDBR470	cortex	right	46, XX	180
11832	Late 8 pcw	HDBR471	midbrain		46, XX	180
11832	Late 8 pcw	HDBR472	medulla oblongata		46, XX	180
11832	Late 8 pcw	HDBR474	diencephalon	left	46, XX	180
11832	Late 8 pcw	HDBR475	cortex	left	46, XX	180
11832	Late 8 pcw	HDBR476	basal ganglia	left	46, XX	180
11832	Late 8 pcw	HDBR478	diencephalon	right	46, XX	180
11832	Late 8 pcw	HDBR479	basal ganglia	right	46, XX	180
11830	Late 8 pcw	HDBR480	cortex	left	46, XX	UNKNOWN
11830	Late 8 pcw	HDBR481	midbrain		46, XX	UNKNOWN
11830	Late 8 pcw	HDBR482	medulla oblongata		46, XX	UNKNOWN
11830	Late 8 pcw	HDBR484	diencephalon	left	46, XX	UNKNOWN
11830	Late 8 pcw	HDBR485	diencephalon	right	46, XX	UNKNOWN
11830	Late 8 pcw	HDBR486	basal ganglia	left	46, XX	UNKNOWN
11830	Late 8 pcw	HDBR487	basal ganglia	right	46, XX	UNKNOWN
11869	Late 8 pcw	HDBR488	midbrain		46, XX	120
11869	Late 8 pcw	HDBR489	hindbrain		46, XX	120
11800	Late 8 pcw	HDBR490	brain fragment		46, XX	120
11492	Late 8 pcw	HDBR491	brain fragment		46, XY	120
1107	16 pcw	HDBR504	cortex slice 1 of 10	right	UNKNOWN	45

1107	16 pcw	HDBR507	cortex slice 4 of 10	right	UNKNOWN	45
1107	16 pcw	HDBR515	cortex slice 9 of 10	right	UNKNOWN	45
1107	16 pcw	HDBR517	cortex slice 3 of 10	right	UNKNOWN	45
1107	16 pcw	HDBR518	cortex slice 4 of 10	right	UNKNOWN	45
11920	CS 23	HDBR519	hindbrain		46, XY	170
11920	CS 23	HDBR520	midbrain		46, XY	170
11920	CS 23	HDBR521	basal ganglia	left	46, XY	170
11920	CS 23	HDBR522	cortex	left	46, XY	170
11920	CS 23	HDBR523	basal ganglia	right	46, XY	170
11907	16 pcw	HDBR524	medulla oblongata	right	46, XX	1000
11907	16 pcw	HDBR525	pons	right	46, XX	1000
11907	16 pcw	HDBR526	midbrain	right	46, XX	1000
11907	16 pcw	HDBR527	temporal lobe slice 1 of 2	right	46, XX	1000
11907	16 pcw	HDBR528	temporal lobe slice 2 of 2	right	46, XX	1000
11907	16 pcw	HDBR529	diencephalon	right	46, XX	1000
11907	16 pcw	HDBR530	choroid plexus	right	46, XX	1000
11907	16 pcw	HDBR531	basal ganglia	right	46, XX	1000
11907	16 pcw	HDBR532	cortex slice 1 of 5	right	46, XX	1000
11907	16 pcw	HDBR533	cortex slice 2 of 5	right	46, XX	1000
11907	16 pcw	HDBR534	cortex slice 3 of 5	right	46, XX	1000
11907	16 pcw	HDBR535	cortex slice 4 of 5	right	46, XX	1000
11907	16 pcw	HDBR536	cortex slice 5 of 5	right	46, XX	1000
11819	16 pcw	HDBR537	brain fragment		46, XX	120
1117	15 pcw	HDBR538	cortex slice 1 of 10	left	UNKNOWN	140
1117	15 pcw	HDBR539	cortex slice 10 of 10	left	UNKNOWN	140
1117	15 pcw	HDBR540	temporal lobe	left	UNKNOWN	140
1117	15 pcw	HDBR541	cortex slice 3 of 10	left	UNKNOWN	140
1117	15 pcw	HDBR542	cortex slice 4 of 10	left	UNKNOWN	140
1117	15 pcw	HDBR543	cortex slice 5 of 10	left	UNKNOWN	140
1117	15 pcw	HDBR544	cortex slice 6 of 10	left	UNKNOWN	140
1117	15 pcw	HDBR545	cortex slice 7 of 10	left	UNKNOWN	140

1117	15 pcw	HDBR546	cortex slice 9 of 10	left	UNKNOWN	140
1290	14 pcw	HDBR547	telencephalon	left	46, XY	1090
11900	14 pcw	HDBR548	hindbrain	left	46, XY	1020
11900	14 pcw	HDBR549	hindbrain	right	46, XY	1020
11900	14 pcw	HDBR550	diencephalon and midbrain		46, XY	1020
11900	14 pcw	HDBR551	temporal lobe	right	46, XY	1020
11900	14 pcw	HDBR552	temporal lobe		46, XY	1020
11900	14 pcw	HDBR553	cortex slice 5 of 5		46, XY	1020
11900	14 pcw	HDBR554	cortex slice 4 of 5		46, XY	1020
11900	14 pcw	HDBR555	cortex slice 2 of 5		46, XY	1020
11900	14 pcw	HDBR556	cortex slice 1 of 5		46, XY	1020
11794	14 pcw	HDBR557	brain fragment		46, XX	120
1923	13 pcw	HDBR558	cortex slice 1 of 3	left	46, XX	150
1923	13 pcw	HDBR559	midbrain		46, XX	150
1923	13 pcw	HDBR560	medulla oblongata		46, XX	150
1923	13 pcw	HDBR561	cortex slice 2 of 3	left	46, XX	150
1923	13 pcw	HDBR562	cortex slice 3 of 3	right	46, XX	150
1923	13 pcw	HDBR563	cortex slice 1 of 2	right	46, XX	150
1923	13 pcw	HDBR564	basal ganglia	left	46, XX	150
1923	13 pcw	HDBR565	basal ganglia	right	46, XX	150
1923	13 pcw	HDBR566	diencephalon	left	46, XX	150
1923	13 pcw	HDBR567	diencephalon	right	46, XX	150
1923	13 pcw	HDBR568	cortex slice 2 of 2	right	46, XX	150
11892	13 pcw	HDBR569	cortex		46, XY	120
11844	13 pcw	HDBR570	forebrain fragment	left	46, XY	120
11501	13 pcw	HDBR571	brain fragment		46, XY	120
1835	12 pcw	HDBR572	cortex slice 1 of 4		46, XY	UNKNOWN
1835	12 pcw	HDBR573	cortex slice 2 of 4		46, XY	UNKNOWN
1835	12 pcw	HDBR574	cortex slice 3 of 4		46, XY	UNKNOWN
1118	12 pcw	HDBR575	temporal lobe slice 1 of 2	right	UNKNOWN	35
11854	12 pcw	HDBR577	midbrain		46, XY	840
11834	12 pcw	HDBR578	cortex slice 1 of 3	right	46, XY	230
11834	12 pcw	HDBR579	midbrain		46, XY	230
11834	12 pcw	HDBR580	hindbrain without cerebellum		46, XY	230
11834	12 pcw	HDBR581	temporal lobe slice 1 of 2	left	46, XY	230
11834	12 pcw	HDBR582	temporal lobe slice 2 of 2	left	46, XY	230
11834	12 pcw	HDBR583	cortex slice 1 of 5	left	46, XY	230
11834	12 pcw	HDBR584	cortex slice 5 of 5	left	46, XY	230

11834	12 pcw	HDBR585	temporal lobe slice 1 of 2	right	46, XY	230
11834	12 pcw	HDBR586	temporal lobe slice 2 of 2	right	46, XY	230
11834	12 pcw	HDBR587	cortex slice 2 of 3	right	46, XY	230
11834	12 pcw	HDBR588	cortex slice 3 of 3	right	46, XY	230
11834	12 pcw	HDBR589	cortex slice 4 of 5	left	46, XY	230
11834	12 pcw	HDBR590	choroid plexus	right	46, XY	230
11834	12 pcw	HDBR591	cortex slice 5 of 5	left	46, XY	230
11834	12 pcw	HDBR592	choroid plexus	left	46, XY	230
11834	12 pcw	HDBR593	diencephalon and pituitary		46, XY	230
1102	12 pcw	HDBR594	forebrain	left	46, XX	200
1102	12 pcw	HDBR595	hindbrain fragmenet		46, XX	200
1558	12 pcw	HDBR596	forebrain	left	46, XX	180
1558	12 pcw	HDBR597	forebrain	right	46, XX	180
1558	12 pcw	HDBR598	midbrain		46, XX	180
11885	12 pcw	HDBR599	cortex slice 1 of 5	left	46, XY	UNKNOWN
11885	12 pcw	HDBR600	cortex slice 5 of 5	left	46, XY	UNKNOWN
11885	12 pcw	HDBR601	temporal lobe slice 2 of 2	left	46, XY	UNKNOWN
11885	12 pcw	HDBR602	cortex slice 1 of 5	right	46, XY	UNKNOWN
11885	12 pcw	HDBR603	cortex slice 5 of 5	right	46, XY	UNKNOWN
11885	12 pcw	HDBR604	temporal lobe	right	46, XY	UNKNOWN
11885	12 pcw	HDBR605	midbrain		46, XY	UNKNOWN
11885	12 pcw	HDBR606	hindbrain		46, XY	UNKNOWN
11885	12 pcw	HDBR607	temporal lobe slice 1 of 2	left	46, XY	UNKNOWN
11885	12 pcw	HDBR608	cortex slice 2 of 5	right	46, XY	UNKNOWN
11885	12 pcw	HDBR609	cortex slice 3 of 5	right	46, XY	UNKNOWN
11885	12 pcw	HDBR610	cortex slice 4 of 5	right	46, XY	UNKNOWN
11885	12 pcw	HDBR611	temporal lobe slice 1 of 2	right	46, XY	UNKNOWN
11885	12 pcw	HDBR612	diencephalon		46, XY	UNKNOWN
1047	12 pcw	HDBR613	brain fragment		46, XX	150
11429	12 pcw	HDBR614	brain fragment		46, XX	120
11602	11 pcw	HDBR615	forebrain	left	46, XY	UNKNOWN
11602	11 pcw	HDBR616	forebrain	right	46, XY	UNKNOWN
11798	11 pcw	HDBR618	brain fragment		46, XY	120
1110	11 pcw	HDBR619	temporal lobe	right	UNKNOWN	30
1123	11 pcw	HDBR620	telencephalon slice 1 of 6	left	UNKNOWN	45
1123	11 pcw	HDBR621	telencephalon slice 3 of 6	left	UNKNOWN	45

1123	11 pcw	HDBR622	telencephalon slice 6 of 6	left	UNKNOWN	45
1123	11 pcw	HDBR623	telencephalon slice 2 of 6	left	UNKNOWN	45
1123	11 pcw	HDBR624	telencephalon slice 4 of 6	left	UNKNOWN	45
1123	11 pcw	HDBR625	telencephalon slice 5 of 6	left	UNKNOWN	45
11656	11 pcw	HDBR626	cortex	left	46, XX	UNKNOWN
11656	11 pcw	HDBR628	midbrain		46, XX	UNKNOWN
11656	11 pcw	HDBR629	basal ganglia		46, XX	UNKNOWN
11609	11 pcw	HDBR630	telencephalon	right	46, XX	UNKNOWN
11609	11 pcw	HDBR632	midbrain		46, XX	UNKNOWN
11833	11 pcw	HDBR633	diencephalon	left	46, XX	1800
11833	11 pcw	HDBR634	midbrain		46, XX	1800
11833	11 pcw	HDBR635	medulla oblongata		46, XX	1800
11833	11 pcw	HDBR638	choroid plexus	left	46, XX	1800
11833	11 pcw	HDBR640	basal ganglia	left	46, XX	1800
11833	11 pcw	HDBR641	choroid plexus	right	46, XX	1800
11833	11 pcw	HDBR642	diencephalon	right	46, XX	1800
11833	11 pcw	HDBR643	temporal lobe	right	46, XX	1800
11833	11 pcw	HDBR644	basal ganglia	right	46, XX	1800
11833	11 pcw	HDBR645	cortex slice 1 of 5	right	46, XX	1800
11833	11 pcw	HDBR646	cortex slice 2 of 5	right	46, XX	1800
11833	11 pcw	HDBR647	cortex slice 3 of 5	right	46, XX	1800
11833	11 pcw	HDBR648	cortex slice 4 of 5	right	46, XX	1800
11833	11 pcw	HDBR649	cortex slice 5 of 5	right	46, XX	1800
11893	11 pcw	HDBR650	forebrain fragment	left	46, XX	120
11806	11 pcw	HDBR651	brain fragment		46, XX	120
11930	11 pcw	HDBR652	cortex slice 1 of 5	right	46, XX	180
11930	11 pcw	HDBR653	cortex slice 5 of 5	right	46, XX	180
11930	11 pcw	HDBR654	temporal lobe slice 1 of 2	right	46, XX	180
11930	11 pcw	HDBR655	diencephalon		46, XX	180
11930	11 pcw	HDBR656	medulla oblongata		46, XX	180
11942	11 pcw	HDBR658	diencephalon	left	46, XX	1000
11942	11 pcw	HDBR659	basal ganglia	left	46, XX	1000
11942	11 pcw	HDBR660	cortex slice 1 of 5	left	46, XX	1000
1890	10 pcw	HDBR661	diencephalon		UNKNOWN	120
1890	10 pcw	HDBR662	choroid plexus		UNKNOWN	120
1281	10 pcw	HDBR663	forebrain	left	46, XY	180
1281	10 pcw	HDBR664	forebrain	right	46, XY	180

1308	10 pcw	HDBR665	forebrain and midbrain		46, XX	120
11841	10 pcw	HDBR668	cortex slice 1 of 5	right	46, XY	120
11841	10 pcw	HDBR669	cortex slice 5 of 5	right	46, XY	120
11841	10 pcw	HDBR670	temporal lobe slice 1 of 2	right	46, XY	120
11841	10 pcw	HDBR671	midbrain		46, XY	120
11841	10 pcw	HDBR672	medulla oblongata		46, XY	120
11841	10 pcw	HDBR673	cortex	left	46, XY	120
11841	10 pcw	HDBR674	basal ganglia	left	46, XY	120
11841	10 pcw	HDBR675	diencephalon	left	46, XY	120
11841	10 pcw	HDBR676	temporal lobe slice 2 of 2	right	46, XY	120
11841	10 pcw	HDBR677	diencephalon	right	46, XY	120
11841	10 pcw	HDBR678	cortex slice 2 of 5	right	46, XY	120
11880	10 pcw	HDBR679	brain fragment		46, XY	120
11947	13 pcw	HDBR691	temporal lobe slice 1 of 2	right	46, XX	500
11947	13 pcw	HDBR692	diencephalon	right	46, XX	500
11947	13 pcw	HDBR693	choroid plexus	right	46, XX	500
11947	13 pcw	HDBR694	basal ganglia	right	46, XX	500
11947	13 pcw	HDBR695	medulla oblongata		46, XX	500
11921	17 pcw	HDBR696	cortex slice 1 of 5	left	46, XY	220
11921	17 pcw	HDBR697	cortex slice 5 of 5	left	46, XY	220
11921	17 pcw	HDBR698	temporal lobe slice 1 of 2	left	46, XY	220
11921	17 pcw	HDBR699	basal ganglia	left	46, XY	220
11921	17 pcw	HDBR700	cortex slice 2 of 5	left	46, XY	220
11122	CS 18	HDBR701	brain fragment		46, XX	120
11238	CS 19	HDBR702	brain fragment		46, XX	120
11286	CS 19	HDBR703	brain fragment		46, XY	120
11284	CS 19	HDBR704	spinal cord		46, XX	120
11287	CS 20	HDBR705	hindbrain		46, XY	120
11237	CS 20	HDBR706	brain fragment		46, XY	120
11382	CS 20	HDBR707	brain fragment		46, XY	120
11254	CS 21	HDBR708	brain fragment		46, XY	120
11471	CS 21	HDBR709	brain fragment		46, XY	120
11811	CS 21	HDBR710	spinal cord		46, XX	120
11849	CS21	HDBR711	spinal cord		46, XY	120
11875	19 pcw	HDBR712	brain fragment		46, XY	120
11882	CS 21	HDBR713	midbrain		46, XX	120
11304	CS 21	HDBR714	brain fragment		46, XX	120
11165	CS 21	HDBR715	brain fragment		46, XX	120
11501	13 pcw	HDBR716	spinal cord		46, XY	120

11814	CS 22	HDBR717	cerebellum	right	46, XY	UNKNOWN
11815	CS 22	HDBR718	cerebellum		46, XX	UNKNOWN
11820	9 pcw	HDBR719	cerebellum	right	46, XX	UNKNOWN
11828	CS 22	HDBR720	cerebellum		46, XX	UNKNOWN
11829	CS 22	HDBR721	cerebellum		46, XY	180
11831	CS 22	HDBR722	cerebellum		46, XY	UNKNOWN
11858	CS 22	HDBR723	cerebellum		46, XY	120
11865	CS 22	HDBR724	cerebellum		46, XY	UNKNOWN
11871	CS 22	HDBR725	cerebellum		46, XX	240
11878	CS 22	HDBR726	cerebellum	right	46, XX	UNKNOWN
1707	CS 22	HDBR727	brain fragment		46, XX	120
1092	CS 22	HDBR728	spinal cord		46, XX	UNKNOWN
11868	CS 22	HDBR729	spinal cord		46, XX	120
11793	CS 22	HDBR730	spinal cord		46, XY	120
11443	CS 22	HDBR731	spinal cord		46, XX	120
11410	CS 22	HDBR732	spinal cord		46, XY	120
1406	CS 23	HDBR733	hindbrain		46, XX	240
1607	CS 23	HDBR734	brain fragment		46, XX	215
11823	CS 23	HDBR735	cerebellum		46, XY	120
11826	CS 23	HDBR736	cerebellum		46, XX	120
11827	CS 23	HDBR737	cerebellum		46, XX	120
11846	CS 23	HDBR738	cerebellum		46, XX	1800
11848	CS 23	HDBR739	hindbrain	right	46, XY	120
11884	CS 23	HDBR740	cerebellum		46, XX	230
1851	CS 23	HDBR741	temporal lobe		UNKNOWN	UNKNOWN
2193	CS 23	HDBR743	brain fragment		46, XY	240
11918	CS 23	HDBR744	cerebellum		46, XX	120
11860	CS 23	HDBR745	spinal cord		46, XX	120
11843	CS 23	HDBR746	spinal cord		46, XX	120
11824	CS 23	HDBR747	spinal cord		46, XY	120
11444	CS 23	HDBR748	spinal cord		46, XY	120
11402	CS 23	HDBR749	spinal cord		46, XX	120
11120	CS 23	HDBR750	brain fragment		46, XY	120
11354	CS 20	HDBR751	brain fragment		46, XY	120
11245	CS 20	HDBR752	brain fragment		46, XX	120
11241	CS 20	HDBR753	brain fragment		46, XX	120
11156	CS 20	HDBR754	brain fragment		46, XY	120
11149	CS 20	HDBR755	hindbrain		46, XX	120
11920	CS 23	HDBR756	diencephalon		46, XY	170
11121	CS 20	HDBR757	cortex		46, XY	120
11118	CS 20	HDBR758	forebrain		46, XX	120
1039	CS 19	HDBR759	spinal cord		46, XX	UNKNOWN
1319	CS 19	HDBR760	hindbrain		46, XX	180
11894	CS 19	HDBR762	brain fragment		46, XY	120
11856	CS 19	HDBR763	spinal cord		46, XY	120
11514	CS 19	HDBR765	spinal cord		46, XX	120

11470	CS 19	HDBR766	brain fragment		46, XX	120
11363	CS 19	HDBR767	brain fragment		46, XX	120
11323	CS 19	HDBR768	brain fragment		46, XX	120
11320	CS 19	HDBR769	brain fragment		46, XY	120
11318	CS 19	HDBR770	brain fragment		46, XY	120
11317	CS 19	HDBR771	brain fragment		46, XY	120
11300	CS 19	HDBR772	brain fragment		46, XY	120
11904	CS 18	HDBR774	hindbrain		46, XY	UNKNOWN
1038	CS 18	HDBR775	spinal cord		46, XY	UNKNOWN
11859	CS 18	HDBR776	brain fragment		46, XY	120
11770	CS 18	HDBR777	spinal cord		46, XX	120
11627	CS 18	HDBR778	spinal cord		46, XX	120
11469	CS 18	HDBR779	brain fragment		46, XX	120
11456	CS 18	HDBR780	spinal cord		46, XX	120
11438	CS 18	HDBR781	brain fragment		46, XY	120
11322	CS 18	HDBR782	brain fragment		46, XY	120
11234	CS 18	HDBR784	brain fragment		46, XX	120
11395	CS 17	HDBR785	brain fragment		46, XY	120
11321	CS 17	HDBR786	brain fragment		46, XY	120
11931	CS 17	HDBR787	spinal cord		UNKNOWN	UNKNOWN
11513	CS 16	HDBR790	spinal cord		46, XY	120
11334	CS 16	HDBR791	brain fragment		46, XX	120
11397	CS 15	HDBR792	brain fragment		46, XX	120
11405	CS 15	HDBR793	brain fragment		46, XX	120
11840	CS 14	HDBR794	hindbrain		46, XX	120
11837	CS 14	HDBR795	hindbrain		46, XY	180
11903	CS 13	HDBR796	midbrain		46, XX	315
11810	9 pcw	HDBR797	hindbrain		46, XX	120
11845	9 pcw	HDBR798	cerebellum		46, XY	120
11851	9 pcw	HDBR799	cerebellum		46, XX	240
11873	9 pcw	HDBR800	cerebellum		46, XX	1080
11606	9 pcw	HDBR802	hindbrain		46, XY	UNKNOWN
11143	CS 20	HDBR803	brain fragment		46, XY	120
11912	9 pcw	HDBR804	cerebellum		46, XY	120
849	9 pcw	HDBR805	spinal cord		46, XX	190
11881	9 pcw	HDBR806	spinal cord		46, XY	120
11874	9 pcw	HDBR807	spinal cord		46, XY	120
11626	9 pcw	HDBR808	Hindbrain		46, XX	120
11312	9 pcw	HDBR809	brain fragment		46, XX	120
11832	Late 8 pcw	HDBR810	cerebellum		46, XX	180
11830	Late 8 pcw	HDBR811	cerebellum		46, XX	UNKNOWN
11869	Late 8 pcw	HDBR812	spinal cord		46, XX	120
11800	Late 8 pcw	HDBR813	spinal cord		46, XX	120
11492	Late 8 pcw	HDBR814	spinal cord		46, XY	120
11160	Late 8 pcw	HDBR815	brain fragment		46, XX	120
11144	Late 8 pcw	HDBR816	brain fragment		46, XY	120

11589	20 pcw	HDBR818	brain fragment		46, XY	120
11876	CS 21	HDBR819	brain fragment		46, XY	120
11581	19 pcw	HDBR820	brain fragment		46, XY	120
1115	17 pcw	HDBR821	temporal lobe (hippocampus)	right	UNKNOWN	35
11808	17 pcw	HDBR822	brain fragment		46, XY	120
11580	17 pcw	HDBR823	brain fragment		46, XY	120
11464	17 pcw	HDBR824	brain fragment		46, XX	120
1107	16 pcw	HDBR825	temporal lobe (hippocampus)	right	UNKNOWN	45
11907	16 pcw	HDBR826	cerebellum	right	46, XX	1000
11819	16 pcw	HDBR827	brain fragment		46, XX	120
1117	15 pcw	HDBR828	temporal lobe	left	UNKNOWN	140
11801	15 pcw	HDBR829	brain fragment		46, XX	120
11446	15 pcw	HDBR831	brain fragment		46, XY	120
1290	14 pcw	HDBR832	diencephalon		46, XY	1090
11900	14 pcw	HDBR833	choroid plexus		46, XY	1020
11794	14 pcw	HDBR836	brain fragment		46, XX	120
11511	14 pcw	HDBR837	brain fragment		46, XY	120
11494	14 pcw	HDBR838	brain fragment		46, XY	120
11457	14 pcw	HDBR839	brain fragment		46, XX	120
11451	14 pcw	HDBR840	brain fragment		46, XY	120
11449	14 pcw	HDBR841	brain fragment		46, XY	120
11424	14 pcw	HDBR842	brain fragment		46, XX	120
1923	13 pcw	HDBR843	cerebellum		46, XX	150
11892	13 pcw	HDBR844	midbrain		46, XY	120
11844	13 pcw	HDBR845	midbrain		46, XY	120
11817	13 pcw	HDBR846	brain fragment		46, XX	120
11775	13 pcw	HDBR847	brain fragment		46, XY	120
11654	13 pcw	HDBR848	brain fragment		46, XX	120
11947	13 pcw	HDBR850	cerebellum		46, XX	500
11496	13 pcw	HDBR851	brain fragment		46, XY	120
11489	13 pcw	HDBR852	brain fragment		46, XX	120
11423	13 pcw	HDBR853	brain fragment		46, XX	120
11349	13 pcw	HDBR854	brain fragment		46, XX	120
11309	13 pcw	HDBR855	brain fragment		46, XX	120
11305	13 pcw	HDBR856	brain fragment		46, XX	120
1835	12 pcw	HDBR857	cortex slice 4 of 4		46, XY	UNKNOWN
1118	12 pcw	HDBR858	temporal lobe slice 2 of 2	right	UNKNOWN	35
11854	12 pcw	HDBR860	cerebellum		46, XY	840
11834	12 pcw	HDBR861	cerebellum		46, XY	230
1102	12 pcw	HDBR862	cerebellum		46, XX	200
1558	12 pcw	HDBR863	hindbrain		46, XX	180
11885	12 pcw	HDBR864	cerebellum		46, XY	UNKNOWN
1047	12 pcw	HDBR865	brain fragment		46, XX	150

1650	12 pcw	HDBR866	spinal cord		46, XX	170
898	12 pcw	HDBR867	spinal cord		46, XX	105
11429	12 pcw	HDBR868	spinal cord		46, XX	120
11373	12 pcw	HDBR869	brain fragment		46, XX	120
11602	11 pcw	HDBR870	cerebellum		46, XY	UNKNOWN
11798	11 pcw	HDBR871	brain fragment		46, XY	120
1110	11 pcw	HDBR872	temporal lobe slice 2 of 2	right	UNKNOWN	30
1123	11 pcw	HDBR873	temporal lobe	left	UNKNOWN	45
1111	11 pcw	HDBR874	temporal lobe	right	UNKNOWN	30
11656	11 pcw	HDBR876	hindbrain		46, XX	UNKNOWN
11609	11 pcw	HDBR877	hindbrain		46, XX	UNKNOWN
11833	11 pcw	HDBR878	cerebellum		46, XX	1800
11787	11 pcw	HDBR881	spinal cord		46, XY	UNKNOWN
1210	11 pcw	HDBR882	spinal cord		46, XX	UNKNOWN
11893	11 pcw	HDBR883	midbrain		46, XX	120
11806	11 pcw	HDBR884	spinal cord		46, XX	120
11769	11 pcw	HDBR885	brain fragment		46, XX	120
11930	11 pcw	HDBR886	cerebellum		46, XX	180
11942	11 pcw	HDBR887	cerebellum		46, XX	1000
1890	10 pcw	HDBR889	medulla oblongata		UNKNOWN	120
1281	10 pcw	HDBR890	hindbrain		46, XY	180
1308	10 pcw	HDBR891	cerebellum		46, XX	120
1046	10 pcw	HDBR892	diencephalon	right	46, XX	120
1036	10 pcw	HDBR893	choroid plexus		46, XX	120
11841	10 pcw	HDBR894	cerebellum		46, XY	120
1263	10 pcw	HDBR896	spinal cord		46, XX	UNKNOWN
1874	10 pcw	HDBR897	spinal cord		UNKNOWN	135
11880	10 pcw	HDBR898	spinal cord		46, XY	120
11573	10 pcw	HDBR899	spinal cord		46, XX	120
11921	17 pcw	HDBR900	cerebellum		46, XY	220
11942	11 pcw	HDBR908	cortex slice 5 of 5	right	46, XX	1000
11666	12 pcw	HDBR909	telencephalon slice 4 of 5	right	46, XY	UNKNOWN
11942	11 pcw	HDBR910	temporal lobe slice 1 of 2	right	46, XX	1000
11942	11 pcw	HDBR911	diencephalon	right	46, XX	1000
11851	9 pcw	HDBR912	cortex slice 3 of 5	left	46, XX	240
11851	9 pcw	HDBR913	cortex slice 2 of 5	left	46, XX	240
11851	9 pcw	HDBR914	cortex slice 4 of 5	left	46, XX	240
11942	11 pcw	HDBR915	temporal lobe slice 1 of 2	left	46, XX	1000
12007	12 pcw	HDBR916	cortex slice 5 of 5	right	46, XY	270
12007	12 pcw	HDBR917	cortex slice 2 of 5	right	46, XY	270
12007	12 pcw	HDBR918	cortex slice 3 of 5	right	46, XY	270
11683	12 pcw	HDBR919	midbrain	left	46, XX	960

12007	12 pcw	HDBR920	diencephalon	right	46, XY	270
12007	12 pcw	HDBR921	choroid plexus	right	46, XY	270
11666	12 pcw	HDBR922	telencephalon slice 2 of 5	right	46, XY	UNKNOWN
11666	12 pcw	HDBR923	telencephalon slice 2 of 5	left	46, XY	UNKNOWN
11666	12 pcw	HDBR924	telencephalon slice 4 of 5	right	46, XY	UNKNOWN
11666	12 pcw	HDBR925	telencephalon slice 4 of 5	left	46, XY	UNKNOWN
11683	12 pcw	HDBR926	telencephalon slice 2 of 5	right	46, XX	960
12007	12 pcw	HDBR927	midbrain		46, XY	270
11683	12 pcw	HDBR928	hindbrain	right	46, XX	960
11683	12 pcw	HDBR929	midbrain	right	46, XX	960
12007	12 pcw	HDBR930	cortex slice 4 of 5	left	46, XY	270
12007	12 pcw	HDBR931	diencephalon	left	46, XY	270
12007	12 pcw	HDBR932	basal ganglia	left	46, XY	270
12007	12 pcw	HDBR933	choroid plexus	left	46, XY	270
11666	12 pcw	HDBR934	hindbrain	left	46, XY	UNKNOWN
11666	12 pcw	HDBR935	hindbrain	right	46, XY	UNKNOWN
11666	12 pcw	HDBR936	midbrain	right	46, XY	UNKNOWN
11653	10 pcw	HDBR937	telencephalon slice 4 of 5	right	46, XY	UNKNOWN
11683	12 pcw	HDBR938	telencephalon slice 4 of 5	right	46, XX	960
11683	12 pcw	HDBR939	telencephalon slice 4 of 5	left	46, XX	960
11930	11 pcw	HDBR940	cortex slice 5 of 5	left	46, XX	180
12007	12 pcw	HDBR941	basal ganglia	right	46, XY	270
11653	10 pcw	HDBR943	telencephalon slice 4 of 5	left	46, XY	UNKNOWN
11653	10 pcw	HDBR944	telencephalon slice 2 of 5	right	46, XY	UNKNOWN
11653	10 pcw	HDBR945	telencephalon slice 2 of 5	left	46, XY	UNKNOWN
11930	11 pcw	HDBR946	cortex slice 3 of 5	left	46, XX	180
11653	10 pcw	HDBR947	midbrain	right	46, XY	UNKNOWN
12007	12 pcw	HDBR948	cortex slice 1 of 5	left	46, XY	270
12007	12 pcw	HDBR949	cortex slice 5 of 5	left	46, XY	270
11942	11 pcw	HDBR950	basal ganglia	right	46, XX	1000
11942	11 pcw	HDBR951	choroid plexus	left	46, XX	1000
11930	11 pcw	HDBR952	cortex slice 4 of 5	left	46, XX	180
11930	11 pcw	HDBR953	cortex slice 2 of 5	left	46, XX	180
11930	11 pcw	HDBR954	cortex slice 2 of 5	right	46, XX	180
11930	11 pcw	HDBR955	cortex slice 3 of 5	right	46, XX	180

11930	11 pcw	HDBR956	cortex slice 4 of 5	right	46, XX	180
11930	11 pcw	HDBR957	cortex slice 1 of 5	left	46, XX	180
11820	9 pcw	HDBR965	cortex slice 3 of 4	right	46, XY	UNKNOWN
11653	10 pcw	HDBR966	midbrain	left	46, XY	UNKNOWN
11666	12 pcw	HDBR967	midbrain	left	46, XY	UNKNOWN
11683	12 pcw	HDBR968	midbrain	left	46, XX	960
12007	12 pcw	HDBR969	cortex slice 1 of 5	right	46, XY	270
