# Supplementary material for: HDBR Expression: A Unique Resource for Global and Individual Gene Expression Studies during Early Human Brain Development
Source: Front Neuroanat. 2016 Oct 26;10:86. doi: 10.3389/fnana.2016.00086 (PMC5080337; doi:10.3389/fnana.2016.00086)
Supplement: Supplementary file 2 [file Table2.pdf]

Supplementary Table 2 **SNP genotyping**

a) details of tissues used for SNP genotyping

Colour	Description
(blue)	RNAseq and SNP genotype data from these samples, RNA and DNA available
(orange)	SNP genotype data from these samples, no RNAseq data, DNA available
(yellow)	SNP genotype data from DNA only samples (to replace samples that failed DNA QC), RNAseq data from other tissues from same individual

HDBR embryo or fetus #	Stage of embryo or fetus	Tissue Sample ID number	tissue morphology of sample	left or right	Karyotype	time sample in transit (minutes)
11793	CS 22	HDBR375	brain fragment		46, XY	120
11849	CS 21	HDBR380	brain fragment		46, XY	120
11566	CS 19	HDBR383	brain fragment		46, XX	120
11626	9 pcw	HDBR469	forebrain fragment		46, XX	120
11122	CS 18	HDBR701	brain fragment		46, XX	120
11238	CS 19	HDBR702	brain fragment		46, XX	120
11286	CS 19	HDBR703	brain fragment		46, XY	120
11287	CS 20	HDBR705	hindbrain		46, XY	120
11237	CS 20	HDBR706	brain fragment		46, XY	120
11382	CS 20	HDBR707	brain fragment		46, XY	120
11254	CS 21	HDBR708	brain fragment		46, XY	120
11471	CS 21	HDBR709	brain fragment		46, XY	120
11811	CS 21	HDBR710	spinal cord		46, XX	120
11875	19 pcw	HDBR712	brain fragment		46, XY	120
11882	CS 21	HDBR713	midbrain		46, XX	120
11304	CS 21	HDBR714	brain fragment		46, XX	120
11165	CS 21	HDBR715	brain fragment		46, XX	120

11501	13 pcw	HDBR716	spinal cord		46, XY	120
11814	CS 22	HDBR717	cerebellum	right	46, XY	UNKNOWN
11815	CS 22	HDBR718	cerebellum		46, XX	UNKNOWN
11828	CS 22	HDBR720	cerebellum		46, XX	UNKNOWN
11829	CS 22	HDBR721	cerebellum		46, XY	180
11831	CS 22	HDBR722	cerebellum		46, XY	UNKNOWN
11858	CS 22	HDBR723	cerebellum		46, XY	120
11865	CS 22	HDBR724	cerebellum		46, XY	UNKNOWN
11871	CS 22	HDBR725	cerebellum		46, XX	240
11878	CS 22	HDBR726	cerebellum	right	46, XX	UNKNOWN
1707	CS 22	HDBR727	brain fragment		46, XX	120
1092	CS 22	HDBR728	spinal cord		46, XX	UNKNOWN
11868	CS 22	HDBR729	spinal cord		46, XX	120
11443	CS 22	HDBR731	spinal cord		46, XX	120
11410	CS 22	HDBR732	spinal cord		46, XY	120
1406	CS 23	HDBR733	hindbrain		46, XX	240
1607	CS 23	HDBR734	brain fragment		46, XX	215
11823	CS 23	HDBR735	cerebellum		46, XY	120
11826	CS 23	HDBR736	cerebellum		46, XX	120
11827	CS 23	HDBR737	cerebellum		46, XX	120
11846	CS 23	HDBR738	cerebellum		46, XX	1800
11848	CS 23	HDBR739	hindbrain	right	46, XY	120
11884	CS 23	HDBR740	cerebellum		46, XX	230
1851	CS 23	HDBR741	temporal lobe		UNKNOWN	UNKNOWN
2193	CS 23	HDBR743	brain fragment		46, XY	240
11918	CS 23	HDBR744	cerebellum		46, XX	120
11860	CS 23	HDBR745	spinal cord		46, XX	120
11843	CS 23	HDBR746	spinal cord		46, XX	120
11824	CS 23	HDBR747	spinal cord		46, XY	120
11444	CS 23	HDBR748	spinal cord		46, XY	120
11402	CS 23	HDBR749	spinal cord		46, XX	120
11120	CS 23	HDBR750	brain fragment		46, XY	120
11354	CS 20	HDBR751	brain fragment		46, XY	120
11245	CS 20	HDBR752	brain fragment		46, XX	120
11241	CS 20	HDBR753	brain fragment		46, XX	120
11156	CS 20	HDBR754	brain fragment		46, XY	120
11149	CS 20	HDBR755	hindbrain		46, XX	120

11920	CS 23	HDBR756	diencephalon		46, XY	170
11121	CS 20	HDBR757	cortex		46, XY	120
11118	CS 20	HDBR758	forebrain		46, XX	120
1039	CS 19	HDBR759	spinal cord		46, XX	UNKNOWN
1319	CS 19	HDBR760	hindbrain		46, XX	180
11894	CS 19	HDBR762	brain fragment		46, XY	120
11856	CS 19	HDBR763	spinal cord		46, XY	120
11514	CS 19	HDBR765	spinal cord		46, XX	120
11470	CS 19	HDBR766	brain fragment		46, XX	120
11363	CS 19	HDBR767	brain fragment		46, XX	120
11323	CS 19	HDBR768	brain fragment		46, XX	120
11320	CS 19	HDBR769	brain fragment		46, XY	120
11318	CS 19	HDBR770	brain fragment		46, XY	120
11317	CS 19	HDBR771	brain fragment		46, XY	120
11300	CS 19	HDBR772	brain fragment		46, XY	120
11904	CS 18	HDBR774	hindbrain		46, XY	UNKNOWN
1038	CS 18	HDBR775	spinal cord		46, XY	UNKNOWN
11859	CS 18	HDBR776	brain fragment		46, XY	120
11770	CS 18	HDBR777	spinal cord		46, XX	120
11627	CS 18	HDBR778	spinal cord		46, XX	120
11469	CS 18	HDBR779	brain fragment		46, XX	120
11456	CS 18	HDBR780	spinal cord		46, XX	120
11322	CS 18	HDBR782	brain fragment		46, XY	120
11288	CS 18	HDBR783	hindbrain	NO RNA-SEQ DATA	UNKNOWN	120
11234	CS 18	HDBR784	brain fragment		46, XX	120
11395	CS 17	HDBR785	brain fragment		46, XY	120
11321	CS 17	HDBR786	brain fragment		46, XY	120
11931	CS 17	HDBR787	spinal cord		UNKNOWN	UNKNOWN

11748	CS 17	HDBR788	hindbrain	NO RNA-SEQ DATA	UNKNOWN	UNKNOWN
11616	CS17	HDBR789	hindbrain	NO RNA-SEQ DATA	46, XX	180
11513	CS 16	HDBR790	spinal cord		46, XY	120
11334	CS 16	HDBR791	brain fragment		46, XX	120
11397	CS 15	HDBR792	brain fragment		46, XX	120
11405	CS 15	HDBR793	brain fragment		46, XX	120
11840	CS 14	HDBR794	hindbrain		46, XX	120
11837	CS 14	HDBR795	hindbrain		46, XY	180
11903	CS 13	HDBR796	midbrain		46, XX	315
11810	9 pcw	HDBR797	hindbrain		46, XX	120
11845	9 pcw	HDBR798	cerebellum		46, XY	120
11851	9 pcw	HDBR799	cerebellum		46, XX	240
1834	12 pcw	HDBR801	midbrain	NO RNA-SEQ DATA	UNKNOWN	UNKNOWN
11606	9 pcw	HDBR802	hindbrain		46, XY	UNKNOWN
11143	CS 20	HDBR803	brain fragment		46, XY	120
849	9 pcw	HDBR805	spinal cord		46, XX	190
11881	9 pcw	HDBR806	spinal cord		46, XY	120
11874	9 pcw	HDBR807	spinal cord		46, XY	120
11312	9 pcw	HDBR809	brain fragment		46, XX	120
11832	Late 8 pcw	HDBR810	cerebellum		46, XX	180
11830	Late 8 pcw	HDBR811	cerebellum		46, XX	UNKNOWN
11869	Late 8 pcw	HDBR812	spinal cord		46, XX	120
11800	Late 8 pcw	HDBR813	spinal cord		46, XX	120
11492	Late 8 pcw	HDBR814	spinal cord		46, XY	120
11160	Late 8 pcw	HDBR815	brain fragment		46, XX	120
11144	Late 8 pcw	HDBR816	brain fragment		46, XY	120
11142	Late 8 pcw	HDBR817	brain fragment	NO RNA-SEQ DATA	46, XY	120
11589	20 pcw	HDBR818	brain fragment		46, XY	120
11876	CS 21	HDBR819	brain fragment		46, XY	120

11581	19 pcw	HDBR820	brain fragment		46, XY	120
1115	17 pcw	HDBR821	temporal lobe (hippocampus)	right	UNKNOWN	35
11808	17 pcw	HDBR822	brain fragment		46, XY	120
11580	17 pcw	HDBR823	brain fragment		46, XY	120
11464	17 pcw	HDBR824	brain fragment		46, XX	120
1107	16 pcw	HDBR825	temporal lobe (hippocampus)	right	UNKNOWN	45
11907	16 pcw	HDBR826	cerebellum	right	46, XX	1000
11819	16 pcw	HDBR827	brain fragment		46, XX	120
1117	15 pcw	HDBR828	temporal lobe	left	UNKNOWN	140
11801	15 pcw	HDBR829	brain fragment		46, XX	120
11484	15 pcw	HDBR830	spinal cord	NO RNA-SEQ DATA	46, XX	120
11446	15 pcw	HDBR831	brain fragment		46, XY	120
1290	14 pcw	HDBR832	diencephalon		46, XY	1090
1836	14 pcw	HDBR834	spinal cord	NO RNA-SEQ DATA	46, XX	UNKNOWN
872	14 pcw	HDBR835	spinal cord	NO RNA-SEQ DATA	46, XY	200
11794	14 pcw	HDBR836	brain fragment		46, XX	120
11511	14 pcw	HDBR837	brain fragment		46, XY	120
11494	14 pcw	HDBR838	brain fragment		46, XY	120
11457	14 pcw	HDBR839	brain fragment		46, XX	120
11451	14 pcw	HDBR840	brain fragment		46, XY	120

11449	14 pcw	HDBR841	brain fragment		46, XY	120
11424	14 pcw	HDBR842	brain fragment		46, XX	120
1923	13 pcw	HDBR843	cerebellum		46, XX	150
11892	13 pcw	HDBR844	midbrain		46, XY	120
11844	13 pcw	HDBR845	midbrain		46, XY	120
11817	13 pcw	HDBR846	brain fragment		46, XX	120
11654	13 pcw	HDBR848	brain fragment		46, XX	120
11572	13 pcw	HDBR849	brain fragment	NO RNA-SEQ DATA	UNKNOWN	120
11947	13 pcw	HDBR850	cerebellum		46, XX	500
11496	13 pcw	HDBR851	brain fragment		46, XY	120
11489	13 pcw	HDBR852	brain fragment		46, XX	120
11423	13 pcw	HDBR853	brain fragment		46, XX	120
11349	13 pcw	HDBR854	brain fragment		46, XX	120
11309	13 pcw	HDBR855	brain fragment		46, XX	120
11305	13 pcw	HDBR856	brain fragment		46, XX	120
1835	12 pcw	HDBR857	cortex slice 4 of 4		46, XY	UNKNOWN
1118	12 pcw	HDBR858	temporal lobe slice 2 of 2	right	UNKNOWN	35
1119	12 pcw	HDBR859	temporal lobe slice 2 of 2	NO RNA-SEQ DATA	UNKNOWN	50
11854	12 pcw	HDBR860	cerebellum		46, XY	840
11834	12 pcw	HDBR861	cerebellum		46, XY	230
1102	12 pcw	HDBR862	cerebellum		46, XX	200
1558	12 pcw	HDBR863	hindbrain		46, XX	180
11885	12 pcw	HDBR864	cerebellum		46, XY	UNKNOWN
1650	12 pcw	HDBR866	spinal cord		46, XX	170
898	12 pcw	HDBR867	spinal cord		46, XX	105
11429	12 pcw	HDBR868	spinal cord		46, XX	120
11373	12 pcw	HDBR869	brain fragment		46, XX	120
11602	11 pcw	HDBR870	cerebellum		46, XY	UNKNOWN

11798	11 pcw	HDBR871	brain fragment		46, XY	120
1110	11 pcw	HDBR872	temporal lobe slice 2 of 2	right	UNKNOWN	30
1123	11 pcw	HDBR873	temporal lobe	left	UNKNOWN	45
1111	11 pcw	HDBR874	temporal lobe	right	UNKNOWN	30
1131	11 pcw	HDBR875	cerebellum	NO RNA-SEQ DATA	UNKNOWN	UNKNOWN
11656	11 pcw	HDBR876	hindbrain		46, XX	UNKNOWN
11609	11 pcw	HDBR877	hindbrain		46, XX	UNKNOWN
11833	11 pcw	HDBR878	cerebellum		46, XX	1800
1116	11 pcw	HDBR879	spinal cord	NO RNA-SEQ DATA	UNKNOWN	60
1845	11 pcw	HDBR880	spinal cord	NO RNA-SEQ DATA	46, XX	1200
11787	11 pcw	HDBR881	spinal cord		46, XY	UNKNOWN
1210	11 pcw	HDBR882	spinal cord		46, XX	UNKNOWN
11893	11 pcw	HDBR883	midbrain		46, XX	120
11806	11 pcw	HDBR884	spinal cord		46, XX	120
11769	11 pcw	HDBR885	brain fragment		46, XX	120
11930	11 pcw	HDBR886	cerebellum		46, XX	180
11942	11 pcw	HDBR887	cerebellum		46, XX	1000
1132	10 pcw	HDBR888	spinal cord	NO RNA-SEQ DATA	UNKNOWN	30
1890	10 pcw	HDBR889	medulla oblongata		UNKNOWN	120
1281	10 pcw	HDBR890	hindbrain		46, XY	180
1308	10 pcw	HDBR891	cerebellum		46, XX	120
1046	10 pcw	HDBR892	diencephalon	right	46, XX	120
11841	10 pcw	HDBR894	cerebellum		46, XY	120
1840	10 pcw	HDBR895	spinal cord	NO RNA-SEQ DATA	UNKNOWN	UNKNOWN
1263	10 pcw	HDBR896	spinal cord		46, XX	UNKNOWN
1874	10 pcw	HDBR897	spinal cord		UNKNOWN	135
11880	10 pcw	HDBR898	spinal cord		46, XY	120
11573	10 pcw	HDBR899	spinal cord		46, XX	120

11873	9 pcw	HDBR958	spinal cord		46, XX	1080
11912	9 pcw	HDBR959	spinal cord		46, XY	120
11900	14 pcw	HDBR960	placenta		46, XY	1020
1406	CS23	HDBR962	lung		46, XX	240
11820	9 pcw	HDBR965	cortex slice 3 of 4	right	46, XY	UNKNOWN
11653	10 pcw	HDBR966	midbrain	left	46, XY	UNKNOWN
11666	12 pcw	HDBR967	midbrain	left	46, XY	UNKNOWN
11683	12 pcw	HDBR968	midbrain	left	46, XX	960
12007	12 pcw	HDBR969	cortex slice 1 of 5	right	46, XY	270

b) details of tissues in wax blocks

	wax block

HDBR embryo or fetus #	Developmental stage of embryo or fetus	Tissue Sample ID number	tissue used for SNP genotyping	tissue in wax block(s)	Karyotype	time sample in transit (minutes)
11577	14 pcw	HDBR001	skin	head	46, XY	240
2197	CS 17	HDBR002	skin	embryo	46, XY	110
2112	CS 22	HDBR003	placenta	head	46, XX	UNKNOWN
2138	CS 22	HDBR004	skin	head, body	46, XX	240
2113	CS 17	HDBR005	placenta	embryo	46, XY	210
2082	CS 22	HDBR006	skin	head	46, XY	90
2089	CS 22	HDBR007	placenta	head, body	46, XX	165
2072	CS 22	HDBR008	skin	head	46, XX	240
2153	CS 17	HDBR009	placenta	embryo,	46, XX	UNKNOWN
1238	CS 21	HDBR010	skin	embryo	46, XY	80
2096	CS 21	HDBR011	placenta	embryo	46, XX	180
2050	CS 22	HDBR012	skin	head, body	46, XX	120
2026	CS 19	HDBR013	skin	embryo	46, XX	120
2144	CS 18	HDBR014	placenta	embryo,	46, XX	115
2145	CS 18	HDBR015	placenta	embryo,	46, XX	130
2191	CS 18	HDBR016	placenta	embryo	46, XX	60
2094	CS 19	HDBR017	skin	embryo	46, XX	UNKNOWN
1248	CS 23	HDBR018	skin	head, body	46, XX	180
1260	CS 23	HDBR019	UNKNOWN	head, body	46, XX	100
1265	CS 23	HDBR020	skin	head, body	46, XY	175

1280	CS 23	HDBR021	skin	head, body	46, XX	180
1778	CS 23	HDBR022	skin	head, body	46, XY	90
1294	CS 23	HDBR023	skin	head, body	46, XX	60
1298	CS 23	HDBR024	skin	head, body	46, XY	120
1301	CS 23	HDBR025	skin	head, body	46, XY	90
1302	CS 23	HDBR026	placenta	head, body	46, XX	60
1310	CS 23	HDBR027	skin	head, body	46, XX	30
1259	CS 23	HDBR028	placenta	head, torso	46, XX	240
1258	CS 23	HDBR029	placenta	head	46, XY	120
1303	CS 22	HDBR031	placenta	head, body	46, XY	180
1306	CS 22	HDBR032	skin	embryo	46, XX	90
1291	CS 21	HDBR033	skin	embryo	46, XY	78
1267	10 pcw	HDBR034	skin	telencephalon	UNKNOWN	110
1300	CS 19	HDBR035	skin	embryo	46, XX	120
1307	CS 19	HDBR036	skin	embryo	46, XY	150
1311	CS 18	HDBR037	placenta	embryo	46, XX	210
1230	CS 14	HDBR038	placenta	embryo	46, XY	240
1254	CS 14	HDBR039	placenta	embryo	46, XX	100
1283	CS 14	HDBR040	placenta	embryo	46, XY	80
1286	CS 14	HDBR041	placenta	embryo	46, XX	110
1282	CS 13	HDBR042	placenta	embryo	46, XY	270
1313	CS 12	HDBR044	skin	embryo	46, XX	120
1234	CS 17	HDBR045	placenta	embryo	46, XY	330
1284	CS 17	HDBR047	placenta	embryo	46, XY	150
1305	CS 15	HDBR048	placenta	embryo	46, XY	75
1315	CS 17	HDBR049	placenta	embryo	46, XY	210
1231	CS 16	HDBR050	placenta	embryo	46, XY	260
1240	CS 16	HDBR051	skin	embryo	46, XX	150
1268	CS 16	HDBR052	placenta	embryo	46, XX	160
1304	CS 16	HDBR053	placenta	embryo	46, XY	280
1334	CS 23	HDBR055	placenta	head, body	46, XY	40
1335	CS 23	HDBR056	placenta	head, body	46, XX	90
1385	CS 23	HDBR057	skin	head	46, XY	150
1408	CS 23	HDBR058	placenta	head, body	46, XX	150
1363	CS 21	HDBR059	placenta	embryo,	46, XY	300

11761	12 pcw	HDBR060	skin	cortex, midbrain, hindbrain	46, XX	UNKNOWN
1339	CS 20	HDBR061	skin	embryo,	46, XY	205
1353	CS 20	HDBR062	skin	embryo,	46, XX	UNKNOWN
1404	CS 20	HDBR064	skin	embryo,	46, XX	360
1324	CS 17	HDBR065	placenta	embryo	46, XX	180
1337	CS 17	HDBR066	skin	embryo,	46, XY	UNKNOWN
1344	CS 17	HDBR067	skin	embryo,	46, XY	85
1402	CS 17	HDBR068	skin	embryo,	46, XX	160
1348	CS 19	HDBR069	skin	embryo,	46, XY	100
1350	CS 19	HDBR070	skin	embryo,	46, XX	180
1360	CS 19	HDBR071	skin	embryo,	46, XY	225
1378	CS 19	HDBR072	skin	embryo,	46, XX	UNKNOWN
1366	CS 18	HDBR073	placenta	embryo,	46, XY	180
1365	CS 15	HDBR074	skin	embryo,	46, XX	90
1374	CS 15	HDBR075	UNKNOWN	embryo,	UNKNOWN	UNKNOWN
1400	CS 15	HDBR076	skin	embryo,	46, XY	114
1356	CS 14	HDBR078	placenta	embryo,	46, XX	70
1379	CS 14	HDBR079	placenta	embryo,	46, XY	270
1340	CS 13	HDBR080	skin	embryo,	46, XY	105
1419	CS 23	HDBR081	skin	head, body	46, XX	180
1458	CS 22	HDBR082	placenta	head, body	46, XY	210
1477	CS 22	HDBR083	skin	head, body	46, XX	165
11855	10 pcw	HDBR084	placenta	head, body	46, XX	800
1452	CS 21	HDBR085	skin	embryo	46, XY	150
1463	CS 21	HDBR086	skin	embryo	46, XY	100
1486	CS 21	HDBR087	skin	embryo	46, XX	205
1465	CS 20	HDBR088	skin	embryo	46, XY	130
1469	CS 22	HDBR089	placenta	embryo	46, XY	70
1414	CS 17	HDBR090	skin	embryo,	46, XX	100
1475	CS 17	HDBR091	skin	embryo	46, XX	260
1489	CS 17	HDBR092	skin	embryo	46, XX	158
1438	CS 16	HDBR093	skin	embryo	46, XX	175
1445	CS 19	HDBR095	placenta	embryo	46, XX	120
1461	CS 18	HDBR096	placenta	embryo	46, XY	150
1480	CS 18	HDBR097	skin	embryo	46, XY	130
1413	CS 14	HDBR098	skin	embryo,	46, XY	90
1447	CS 14	HDBR100	placenta	embryo	46, XX	270
1485	CS 14	HDBR101	skin	embryo	46, XX	145
1490	CS 14	HDBR102	skin	embryo	46, XX	145

1434	CS 13	HDBR103	placenta	embryo	46, XX	150
1446	CS 13	HDBR104	placenta	embryo	46, XX	270
1506	CS 13	HDBR105	skin	embryo	46, XY	180
1507	CS 13	HDBR106	skin	embryo	46, XY	UNKNOWN
1497	9 pcw	HDBR109	skin	brain	46, XY	120
1444	11 pcw	HDBR110	skin	cortex	46, XX	170
1555	CS 22	HDBR111	skin	head, body	46, XY	300
1537	CS 17	HDBR112	placenta	embryo	46, XY	90
1560	CS 20	HDBR121	placenta	embryo	46, XY	180
1574	CS 19	HDBR122	placenta	embryo	46, XX	270
1579	CS 19	HDBR123	skin	embryo	46, XX	220
1586	CS 19	HDBR124	skin	embryo	46, XX	106
1568	CS 16	HDBR129	placenta	embryo	46, XX	150
1621	CS 22	HDBR131	skin	head, body	46, XX	110
1640	CS 22	HDBR132	skin	embryo	46, XX	120
1663	CS 15	HDBR134	placenta	embryo	UNKNOWN	400
1608	CS 19	HDBR135	skin	embryo	46, XX	270
1670	CS 18	HDBR136	placenta	embryo	46, XX	180
1661	CS 16	HDBR137	placenta	embryo	46, XY	270
1697	CS 16	HDBR138	skin	embryo,	46, XY	80
1698	CS 16	HDBR139	skin	embryo,	46, XX	210
1635	CS 14	HDBR141	skin	embryo	46, XX	310
1722	CS 17	HDBR142	skin	embryo,	46, XY	180
1758	CS 17	HDBR143	skin	embryo	46, XX	120
1731	CS 17	HDBR144	placenta	embryo	46, XY	120
1720	CS 16	HDBR145	skin	embryo,	46, XX	180
1787	CS 19	HDBR146	skin	embryo	46, XX	150
1775	CS 14	HDBR147	skin	embryo	UNKNOWN	170
1826	CS 22	HDBR148	placenta	head, body	46, XY	UNKNOWN
1872	CS 22	HDBR149	skin	head, body	UNKNOWN	180
1894	CS 17	HDBR150	placenta	embryo	46, XY	180
1895	CS 16	HDBR151	placenta	embryo	46, XY	240
1848	CS 19	HDBR152	skin	embryo	UNKNOWN	270
1944	9 pcw	HDBR153	skin	brain	46, XY	270
1967	CS 22	HDBR154	skin	head, body	46, XY	90
1999	CS 22	HDBR155	skin	head	46, XY	120
1937	CS 21	HDBR156	placenta	embryo,	46, XY	UNKNOWN
1972	CS 21	HDBR157	skin	embryo	46, XY	270
1979	CS 21	HDBR158	skin	embryo	46, XY	185
1929	CS 20	HDBR159	skin	embryo,	46, XY	240
1936	CS 20	HDBR160	placenta	embryo,	46, XY	UNKNOWN
2008	CS 18	HDBR161	placenta	embryo	46, XY	UNKNOWN

1921	CS 17	HDBR162	skin	embryo,	UNKNOWN	135
1905	CS 16	HDBR163	skin	embryo	46, XX	150
1906	CS 16	HDBR164	skin	embryo	46, XY	240
1911	CS 16	HDBR165	skin	embryo	46, XX	105
1913	CS 16	HDBR166	skin	embryo	46, XY	240
1931	CS 16	HDBR167	placenta	embryo,	46, XY	180
1948	CS 16	HDBR168	skin	embryo	46, XX	150
1962	CS 16	HDBR169	skin	embryo	46, XX	240
1978	CS 16	HDBR170	skin	embryo	46, XY	120
1980	CS 16	HDBR171	skin	embryo	46, XY	270
1917	CS 14	HDBR172	placenta	embryo	46, XX	330
1964	CS 14	HDBR173	skin	embryo	46, XY	180
1908	9 pcw	HDBR174	skin	brain	46, XY	120
1242	11 pcw	HDBR175	skin	telencep halon	46, XY	135
1246	CS 12	HDBR176	placenta	embryo	46, XY	110
1317	10 pcw	HDBR177	skin	telencep halon hindbrain	UNKNOWN	1080
1318	11 pcw	HDBR178	skin	telencep halon	46, XX	UNKNOWN
1333	11 pcw	HDBR179	skin	telencep halon	46, XX	60
1430	13 pcw	HDBR180	skin	cortex	46, XY	120
1492	11 pcw	HDBR182	skin	cortex	46, XX	150
1493	13 pcw	HDBR183	skin	cortex	46, XY	240
1552	10 pcw	HDBR185	skin	cortex	46, XY	135
1565	11 pcw	HDBR186	skin	cortex, cerebellu m	46, XY	120
1578	CS 15	HDBR187	skin	embryo	46, XX	230
1610	Late 8 pcw	HDBR188	placenta	head, body	46, XX	160
1611	Late 8 pcw	HDBR189	placenta	head, body	46, XY	37
1650	12 pcw	HDBR190	skin	cortex	46, XX	170
1665	11 pcw	HDBR191	skin	cortex	46, XX	150
1666	9 pcw	HDBR192	skin	head	46, XX	180
1729	Late 8 pcw	HDBR193	skin	head, body	46, XX	150
1829	12 pcw	HDBR194	placenta	embryo	46, XY	120
1868	Late 8 pcw	HDBR195	skin	head	UNKNOWN	180
1889	Late 8 pcw	HDBR196	skin	brain (no olfactory bulbs)	46, XX	UNKNOWN

1893	9 pcw	HDBR197	skin	forebrain, midbrain, hindbrain	46, XY	120
1916	Late 8 pcw	HDBR198	skin	head, body	46, XY	300
1946	11 pcw	HDBR199	skin	cortex	46, XY	1380
1949	CS 20	HDBR200	skin	embryo	UNKNOWN	90
1950	Late 8 pcw	HDBR201	skin	head, body	UNKNOWN	110
1966	11 pcw	HDBR202	skin	cortex	46, XY	180
1987	Late 8 pcw	HDBR203	skin	head, body	46, XX	60
2172	12 pcw	HDBR204	skin	cortex, hindbrain	46, XY	185
2173	12 pcw	HDBR205	skin	cortex, cerebellum	46, XX	210
2184	Late 8 pcw	HDBR206	skin	head, body	46, XY	600
2185	Late 8 pcw	HDBR207	skin	head, body	46, XY	600
2198	9 pcw	HDBR208	skin	head	46, XY	100
2015	CS 20	HDBR209	placenta	embryo	46, XY	240
2036	10 pcw	HDBR210	skin	cortex	46, XX	140
2037	13 pcw	HDBR211	skin	cortex	46, XY	160
2045	13 pcw	HDBR212	skin	cortex	46, XX	120
2046	10 pcw	HDBR213	skin	cortex	46, XX	180
2051	CS 20	HDBR214	placenta	embryo	46, XX	1170
2064	Late 8 pcw	HDBR215	skin	head, body	46, XY	1380
11631	Late 8 pcw	HDBR216	skin	head	46, XY	UNKNOWN
11680	Late 8 pcw	HDBR217	skin	head, body	46, XY	UNKNOWN
11705	Late 8 pcw	HDBR218	skin	head, body	46, XY	1200
11719	Late 8 pcw	HDBR219	skin	head, body	46, XX	120
1675	13 pcw	HDBR220	skin	cortex	46, XY	210
2038	CS 17	HDBR221	skin	embryo	46, XX	245
2041	CS 16	HDBR222	placenta	embryo	46, XX	90
2090	CS 17	HDBR223	placenta	embryo	46, XX	130
2127	CS 21	HDBR224	placenta	embryo	46, XX	UNKNOWN
2129	CS 21	HDBR225	placenta	embryo	46, XY	120
2130	CS 16	HDBR226	skin	embryo	46, XY	105

2177	10 pcw	HDBR227	skin	cortex, hindbrain	UNKNOWN	90
2178	CS 21	HDBR228	skin	embryo	46, XY	175
2187	CS 19	HDBR229	skin	embryo	46, XY	210
2181	CS 22	HDBR230	skin	embryo	46, XY	60
11682	CS 23	HDBR231	skin	head	46, XY	90
11717	CS 22	HDBR232	skin	head, body	46, XX	130
11762	10 pcw	HDBR233	skin	cortex, midbrain, hindbrain	46, XY	UNKNOWN
11670	CS 23	HDBR234	placenta	head, body	46, XY	90
11649	CS 23	HDBR235	skin	head, body	46, XY	90
11608	CS 23	HDBR236	skin	brain	46, XY	UNKNOWN
1449	CS 13	HDBR237	placenta	embryo	46, XY	240
1481	9 pcw	HDBR238	skin	cortex	46, XX	125
1513	11 pcw	HDBR240	skin	cortex	46, XY	240
1575	CS 13	HDBR241	placenta	embryo	46, XY	200
11700	9 pcw	HDBR242	skin	brain	46, XY	UNKNOWN
1563	CS 13	HDBR243	placenta	embryo	46, XX	180
1617	CS 23	HDBR244	skin	head, body	46, XY	190
1724	12 pcw	HDBR245	skin	cortex	46, XX	85
1744	CS 23	HDBR246	skin	head, body	46, XY	300
1745	CS 23	HDBR247	skin	head, body	46, XX	180
1786	CS 22	HDBR248	skin	embryo	46, XX	174
1777	CS 23	HDBR249	skin	head, body	46, XY	120
1918	CS 23	HDBR250	placenta	head, body	46, XY	450
11867	CS17	HDBR901	placenta	embryo	46, XY	UNKNOWN
11674	CS12	HDBR902	UNKNOWN	embryo	46, XX	80
11913	9 pcw	HDBR903	placenta	head, body	46, XX	720
11723	CS21	HDBR904	UNKNOWN	embryo	46, XY	120
2195	CS14	HDBR905	UNKNOWN	embryo	46, XX	210
11712	CS21	HDBR906	placenta	embryo	46, XY	120
11735	CS15	HDBR907	placenta	embryo	46, XY	180
