# Supplementary material for: HDBR Expression: A Unique Resource for Global and Individual Gene Expression Studies during Early Human Brain Development
Source: Front Neuroanat. 2016 Oct 26;10:86. doi: 10.3389/fnana.2016.00086 (PMC5080337; doi:10.3389/fnana.2016.00086)
Supplement: Supplementary file 3 [file Table3.pdf]

Supplementary Table 3 **Top 200 genes differentially expressed between 9-12 PCW**

Top 200 protein coding genes that are differentially expressed between 9PCW and 12 PCW with the lowest p value (fold change >2)

Gene Symbol	Log 2 fold	P adjusted
HDHD1	1.036406213	2.82E-39
DYNC1I1	-1.937347079	5.30E-38
BCAS1	-4.21646826	1.62E-31
RASL10A	-3.855068264	1.07E-30
CIDEA	-4.035745122	2.63E-28
LIN28B	2.865437872	9.06E-28
PSTPIP2	1.247049312	9.06E-28
TMOD1	-2.903499997	9.06E-28
SCD5	-1.419099014	1.08E-27
MAFF	-1.654376105	1.51E-27
HTR1A	-3.810910701	3.03E-26
HSPA6	-4.789756785	3.03E-26
SLCO1C1	-3.035869614	6.57E-26
GPX3	-3.330593594	2.99E-25
ZNF658	1.03244712	5.36E-25
ACY3	-2.991134193	1.01E-24
LARGE	-1.23692116	1.05E-24
TMEM74	-1.359305117	2.67E-24
STARD5	-1.687079232	4.96E-24
HIF3A	1.596976634	1.06E-23
SERPINE1	-2.320634042	1.09E-23
LIMCH1	-1.872800611	1.41E-23
NDRG1	-2.002482897	5.40E-23
NUDT13	1.244054419	2.66E-22
GADD45B	-2.461291738	1.15E-21
WASF1	-1.504638348	1.22E-21
HSPA1A	-3.059663682	2.62E-21
CYR61	-1.886434695	3.33E-21
LPL	-2.473995173	4.36E-21
PTPRQ	4.295245357	1.47E-20
CRYM	-3.163538495	1.81E-20
DUSP1	-1.129791759	4.16E-20
KCTD12	-1.68006269	9.15E-20
ITGB5	-2.797474446	9.37E-20
SRSF6	1.0743457	9.58E-20
RGS6	-2.422529019	1.04E-19
NHS	1.846459295	1.10E-19
NDST4	2.446380774	3.46E-19
SLC35F2	-2.327910539	3.68E-19
EDN1	-1.383276716	4.45E-19
TMEM108	-1.628263585	4.88E-19

HS3ST4	-2.048937259	1.22E-18
INHBA	-3.050150611	1.22E-18
TTLL2	-2.705766923	1.66E-18
TMEM159	-1.438084747	4.96E-18
C1orf95	-1.919960968	1.35E-17
GAB2	-1.377893767	1.60E-17
APIP	1.141853264	1.83E-17
ATP2C2	-2.685291763	1.83E-17
SYTL4	1.118449158	1.97E-17
FAM110B	-1.029880659	1.99E-17
DIRAS3	2.436985305	4.15E-17
SEMA7A	-1.925202754	5.50E-17
HSPA1B	-2.63453646	7.49E-17
FAM149A	-1.098434915	7.64E-17
OTOR	-3.637188584	8.39E-17
KRT31	-4.120775404	1.52E-16
DDIT4	-1.599122088	2.31E-16
FLRT1	-1.381080465	2.36E-16
NCK2	-1.084683862	2.37E-16
SCN11A	1.181506407	3.04E-16
ADAMTS5	2.108111086	3.19E-16
CCNJ	1.059928847	4.60E-16
ADM	-1.716435374	5.53E-16
SV2C	2.546574702	8.47E-16
FAM227A	1.09627181	9.40E-16
SLC26A2	1.146537114	9.80E-16
TBC1D8B	1.637220293	1.04E-15
AGAP1	-1.422589131	1.04E-15
CRYGS	1.282597393	1.54E-15
HTR2B	-2.826589998	1.82E-15
EYA2	2.308299509	1.86E-15
TPH1	1.646422971	2.41E-15
SIK1	-1.333134736	2.51E-15
SH2D2A	-1.931472479	2.52E-15
SOWAHB	-1.960087246	2.52E-15
KRT34	-4.016801799	2.53E-15
LDB2	-1.390108841	4.38E-15
ACRC	1.342084915	5.56E-15
SPINK5	3.144914762	9.09E-15
C3	-1.58772598	1.07E-14
PHLDB2	1.625391163	1.17E-14
AHNAK2	-1.357824211	1.24E-14
CCDC173	1.480312709	1.63E-14
ADAMTSL1	-1.768522459	1.63E-14
JUN	-1.651705159	1.81E-14
SLC9C2	1.337764398	1.93E-14
ENOSF1	1.812963208	2.11E-14
IGSF3	-1.171048211	2.35E-14
B3GALT2	-1.337461195	2.46E-14

MAT2A	1.07080628	2.62E-14
SYT10	2.253876006	3.09E-14
TDRD7	-1.166108991	3.12E-14
GRM3	-1.733214717	3.81E-14
SOBP	-1.624788977	4.00E-14
PRSS23	-1.15678247	5.01E-14
NR4A2	-1.959099345	8.01E-14
CLCN5	1.531298943	8.07E-14
APLN	-1.353922846	9.11E-14
PLCH1	1.243028579	9.36E-14
DDX26B	1.187741516	1.01E-13
HMGA2	2.42882644	1.02E-13
GABRP	2.086788024	1.04E-13
IL27RA	-1.582028049	1.16E-13
PDZD7	-1.646697369	1.46E-13
ME3	-1.058124764	1.77E-13
LYPD5	-1.054422089	1.81E-13
FAM101B	-1.518205101	1.96E-13
CCND1	1.97263006	2.26E-13
ANKHD1-EIF4EBP3	1.327599442	2.30E-13
GPR50	2.855073641	2.54E-13
RTN4RL1	-1.75920198	2.61E-13
MAPT	-1.721959135	2.69E-13
SCNN1G	-2.124410007	2.84E-13
TUBB2B	-1.085178153	3.69E-13
SMOC1	-1.739225078	5.08E-13
MMRN1	1.594108018	5.50E-13
CHRM5	-3.135105025	5.69E-13
CCL3	-2.567332649	6.32E-13
GABRE	1.62782374	6.35E-13
ATP1A1	-1.014127683	7.83E-13
KIF26B	-2.026416883	8.34E-13
NXPH3	-2.421847619	8.38E-13
PPFIBP2	-1.125915165	8.59E-13
LRP5L	-1.336537205	9.75E-13
PTGDR2	-2.128118729	1.10E-12
TLR3	-1.586177556	1.13E-12
CASP1	-1.684565373	1.14E-12
MTUS2	-1.385670095	1.24E-12
ZNF730	1.537212974	1.30E-12
CCL3L3	-2.770310379	1.31E-12
SH3KBP1	-1.073934725	1.40E-12
ASTN2	-1.752090772	1.48E-12
ERICH5	-2.323898407	1.50E-12
RND1	-1.248056037	1.52E-12
TRIM67	-1.509887841	1.56E-12
TAP1	-1.955396556	1.69E-12
MAP3K15	-1.399503664	1.84E-12

PLAG1	1.794090145	1.89E-12
IFI44L	2.000428647	1.98E-12
NODAL	-1.414278922	2.12E-12
FPR1	-1.769487583	2.37E-12
FAP	2.747006322	2.43E-12
TMEM178A	-1.246773516	2.45E-12
HLA-C	-2.045282181	2.45E-12
FOS	-1.882460041	2.51E-12
NELL2	-1.116779016	2.60E-12
SLA	-1.675145546	2.71E-12
STMN4	-1.557584041	2.81E-12
KIAA1217	1.645829508	2.93E-12
SHISA9	-1.881593626	2.96E-12
B3GALT5	1.466806477	3.30E-12
ADRB1	-2.487501461	3.30E-12
ADD3	1.280180859	3.37E-12
UBE2QL1	-1.571338991	3.78E-12
GDPD5	-1.746077869	4.64E-12
KCNK9	-2.243579859	5.13E-12
SLC25A25	-1.113362558	5.14E-12
ZC3H12C	1.233895254	5.18E-12
LCOR	1.131537186	5.21E-12
Mar-03	-1.599489806	5.21E-12
TSHZ3	-1.868857349	5.63E-12
CNTNAP2	1.526591651	6.29E-12
LGALS3	-1.9403598	6.29E-12
KRT33B	-3.500151694	7.06E-12
IGF2BP1	1.22902062	7.90E-12
CSF1R	-1.576729901	8.05E-12
MYL4	-2.189925097	8.78E-12
RHCG	-1.932235479	9.03E-12
LHX9	2.101447191	1.04E-11
RNF113A	-1.263894342	1.05E-11
MGAM	1.461266409	1.15E-11
FAM196A	-1.568621365	1.21E-11
HEPACAM	-2.879262838	1.23E-11
HMGN5	1.349115257	1.36E-11
PCDH11Y	-2.10475491	1.49E-11
L1TD1	1.943111035	1.62E-11
LARP6	-1.005320705	1.85E-11
TRIM66	1.499279415	2.17E-11
CCDC14	1.120656137	2.28E-11
EVA1C	-1.86785476	2.37E-11
NKX2-2	-2.144896738	2.63E-11
DSEL	-1.346588268	2.80E-11
ALS2CR12	1.190349976	3.10E-11
HHIPL1	-1.108253586	3.12E-11
CLMN	1.575820013	4.11E-11
KLHL31	1.444083098	4.14E-11

SEMA3C	-1.279306443	4.23E-11
PIK3CD	-1.220634494	4.98E-11
ADAMTS19	1.768334633	5.35E-11
CMTR2	1.035337739	5.61E-11
TEX15	1.764082704	6.25E-11
HLA-B	-1.760599387	6.25E-11
ABCC2	1.262102656	6.58E-11
ZNF518A	1.074205262	7.37E-11
AOAH	-1.187621733	7.98E-11
SELPLG	-1.517286364	8.64E-11
CDC42EP1	-2.173740896	8.70E-11
FOSL2	-2.080639434	8.87E-11
PDXK	-1.166511554	9.17E-11

Top 200 protein coding genes that are differentially expressed between 9PCW and 12 PCW - with the highest fold change (p adjusted <0.05)

Gene Name	Log 2 fold	P adjusted
HSPA6	4.789757	3.03E-26
PTPRQ	4.295245	1.47E-20
BCAS1	4.216468	1.62E-31
KRT31	4.120775	1.52E-16
CIDEA	4.035745	2.63E-28
KRT34	4.016802	2.53E-15
RASL10A	3.855068	1.07E-30
HTR1A	3.810911	3.03E-26
OTOR	3.637189	8.39E-17
KRT33B	3.500152	7.06E-12
ZFP57	3.47717	1.09E-10
GPX3	3.330594	2.99E-25
AQP4	3.253096	1.20E-10
CRYM	3.163538	1.81E-20
KRT33A	3.152875	1.38E-08
SPINK5	3.144915	9.09E-15
CHRM5	3.135105	5.69E-13
HSPA1A	3.059664	2.62E-21
INHBA	3.050151	1.22E-18
SLCO1C1	3.03587	6.57E-26
ACY3	2.991134	1.01E-24
DEFB112	2.978308	3.62E-09
TMOD1	2.9035	9.06E-28
HEPACAM	2.879263	1.23E-11
LIN28B	2.865438	9.06E-28
GPR50	2.855074	2.54E-13
HTR2B	2.82659	1.82E-15
KRT19	2.815798	2.40E-10

ITGB5	2.797474	9.37E-20
CCL3L3	2.77031	1.31E-12
MAG	2.761447	2.88E-07
FAP	2.747006	2.43E-12
CD2	2.731503	1.17E-06
DAZ2	2.726465	1.52E-06
TTLL2	2.705767	1.66E-18
ATP2C2	2.685292	1.83E-17
HSPA1B	2.634536	7.49E-17
C3orf80	2.609697	1.12E-08
DAZ1	2.59296	5.87E-06
TAAR9	2.582372	3.56E-07
CCL3	2.567333	6.32E-13
SV2C	2.546575	8.47E-16
OSM	2.504266	1.57E-10
TMC3	2.501266	4.65E-07
DAZ3	2.492306	1.46E-05
ADRB1	2.487501	3.30E-12
LPL	2.473995	4.36E-21
CRYAB	2.46982	2.14E-06
EBF2	2.463912	3.90E-10
GADD45B	2.461292	1.15E-21
ADIRF	2.455737	1.65E-05
NDST4	2.446381	3.46E-19
GPR17	2.437764	1.42E-08
DIRAS3	2.436985	4.15E-17
HMGA2	2.428826	1.02E-13
RAMP3	2.42796	8.47E-08
RGS6	2.422529	1.04E-19
NXPH3	2.421848	8.38E-13
OSTN	2.416467	6.48E-10
HTR3A	2.411307	6.04E-07
FA2H	2.377963	1.42E-06
VSX1	2.340006	2.52E-09
IL12RB2	2.329874	1.84E-06
SLC35F2	2.327911	3.68E-19
ERICH5	2.323898	1.50E-12
SERPINE1	2.320634	1.09E-23
EYA2	2.3083	1.86E-15
AGT	2.304714	3.84E-05
SULT1E1	2.303907	1.10E-08
PAPLN	2.292464	1.24E-10
KRT13	2.287289	1.40E-06
SYT10	2.253876	3.09E-14
KCNK9	2.24358	5.13E-12
FGF10	2.241131	9.40E-06
IQCJ-SCHIP1	2.221233	2.64E-07
CH25H	2.220792	1.28E-08
UNCX	2.20735	5.48E-07

MYL4	2.189925	8.78E-12
TNC	2.184324	4.14E-09
MOG	2.183881	1.41E-06
SULT1C2	2.181035	1.23E-05
TBX21	2.180791	0.000171936
FAM163A	2.174863	6.15E-07
CDC42EP1	2.173741	8.70E-11
LCE5A	2.163304	8.22E-05
MT1M	2.157847	1.39E-05
SLN	2.148791	5.89E-09
NKX2-2	2.144897	2.63E-11
FRMPD4	2.139676	9.74E-10
ARHGAP36	2.137153	6.11E-07
PTGDR2	2.128119	1.10E-12
SCNN1G	2.12441	2.84E-13
TMEM102	2.111661	3.40E-09
SIX2	2.110486	0.000181156
BBOX1	2.108356	4.57E-05
ADAMTS5	2.108111	3.19E-16
KRT32	2.108022	0.000174529
PCDH11Y	2.104755	1.49E-11
LHX9	2.101447	1.04E-11
SPRR2D	2.095641	0.000265192
TLR2	2.095366	1.79E-08
CRYGC	2.093131	6.55E-05
GABRP	2.086788	1.04E-13
SIX1	2.086667	2.12E-05
FOSL2	2.080639	8.87E-11
GLRA2	2.079189	3.01E-10
HAPLN4	2.07628	3.44E-05
SATB2	2.070723	3.07E-08
CLDN23	2.068993	3.19E-06
BNC2	2.064444	5.30E-06
HS3ST4	2.048937	1.22E-18
F11	2.045579	0.000317099
HLA-C	2.045282	2.45E-12
HRH2	2.04192	2.28E-10
LIX1	2.03841	3.79E-10
FFAR3	2.037553	0.000357933
FRMD7	2.035523	0.000139582
CDH7	2.0326	1.15E-07
DCDC2C	2.0304	5.24E-05
KIF26B	2.026417	8.34E-13
DMRTB1	2.024068	0.000209034
ZFP42	2.02342	0.000313891
DEFA1B	2.023142	0.000528869
C10orf82	2.021508	6.39E-09
GPAT2	2.018198	2.67E-07
NDRG1	2.002483	5.40E-23

PSMB9	2.00132	7.43E-06
IFI44L	2.000429	1.98E-12
TUBA3D	1.996973	0.000448678
AC011155.1	1.990526	4.30E-06
LMX1B	1.990156	0.000515944
DLK1	1.983053	6.65E-05
WFDC1	1.975597	3.88E-06
CCND1	1.97263	2.26E-13
RPRM	1.964752	6.11E-10
SOWAHB	1.960087	2.52E-15
NR4A2	1.959099	8.01E-14
HSPA12A	1.95812	3.17E-08
TAP1	1.955397	1.69E-12
GSTM1	1.951048	1.80E-09
L1TD1	1.943111	1.62E-11
OTOL1	1.941835	0.001051675
LGALS3	1.94036	6.29E-12
DYNC1I1	1.937347	5.30E-38
GADL1	1.936371	7.55E-05
RHCG	1.932235	9.03E-12
SH2D2A	1.931472	2.52E-15
CFH	1.926696	6.22E-06
SEMA7A	1.925203	5.50E-17
OLAH	1.923195	1.90E-05
LCN2	1.922863	0.00033607
HKDC1	1.921033	1.51E-08
CSF3	1.920389	0.000116954
C1orf95	1.919961	1.35E-17
C6orf141	1.911174	1.68E-05
MPZL2	1.9057	0.000800295
RXRG	1.90109	0.000385838
NAPRT	1.892101	1.11E-06
C2CD4B	1.891664	3.65E-10
MYH4	1.889875	0.000391397
SLC44A4	1.889602	1.45E-05
CYR61	1.886435	3.33E-21
FOS	1.88246	2.51E-12
SHISA9	1.881594	2.96E-12
NEFM	1.880194	5.71E-08
CRABP1	1.880164	5.96E-06
GFY	1.872993	1.68E-05
LIMCH1	1.872801	1.41E-23
TSHZ3	1.868857	5.63E-12
EVA1C	1.867855	2.37E-11
G0S2	1.855041	0.00051163
PRPS1L1	1.852949	0.000309747
LRRTM1	1.851177	6.78E-08
NHS	1.846459	1.10E-19
OR5M8	1.842173	0.001093831

OGN	1.841665	0.00186276
LEF1	1.839851	1.13E-09
DEFB110	1.83813	0.001796698
VHLL	1.83707	9.53E-06
COL6A3	1.833995	0.000999464
HGF	1.829095	1.08E-08
SCRG1	1.826111	3.58E-06
FRMPD2	1.821707	6.45E-05
C8orf34	1.81811	7.08E-10
ENOSF1	1.812963	2.11E-14
TSHZ2	1.808606	1.73E-05
LUM	1.805274	6.89E-05
TAS2R10	1.80082	2.14E-09
GPR64	1.796264	2.82E-05
HSPB1	1.795849	1.37E-05
PLAG1	1.79409	1.89E-12
SALL4	1.788668	5.73E-10
DNAJB1	1.784406	3.05E-10
C7	1.780551	0.001501459
PLD4	1.780056	2.83E-10
CPNE8	1.779081	3.57E-05
DEFA3	1.776043	0.002779504
CPLX1	1.775468	8.61E-08
TRPC6	1.769692	7.25E-08
FPR1	1.769488	2.37E-12

81 genes that are present in both the highest fold change and lowest p value lists

Gene Symbol	Log 2 fold	P adjusted
ACY3	2.991134193	1.01E-24
ADAMTS5	-2.108111086	3.19E-16
ADRB1	2.487501461	3.30E-12
ATP2C2	2.685291763	1.83E-17
BCAS1	4.21646826	1.62E-31
C1orf95	1.919960968	1.35E-17
CCL3	2.567332649	6.32E-13
CCL3L3	2.770310379	1.31E-12
CCND1	-1.97263006	2.26E-13
CDC42EP1	2.173740896	8.70E-11
CHRM5	3.135105025	5.69E-13
CIDEA	4.035745122	2.63E-28
CRYM	3.163538495	1.81E-20
CYR61	1.886434695	3.33E-21
DIRAS3	-2.436985305	4.15E-17
DYNC1I1	1.937347079	5.30E-38
ENOSF1	-1.812963208	2.11E-14

ERICH5	2.323898407	1.50E-12
EVA1C	1.86785476	2.37E-11
EYA2	-2.308299509	1.86E-15
FAP	-2.747006322	2.43E-12
FOS	1.882460041	2.51E-12
FOSL2	2.080639434	8.87E-11
FPR1	1.769487583	2.37E-12
GABRP	-2.086788024	1.04E-13
GADD45B	2.461291738	1.15E-21
GPR50	-2.855073641	2.54E-13
GPX3	3.330593594	2.99E-25
HEPACAM	2.879262838	1.23E-11
HLA-C	2.045282181	2.45E-12
HMGA2	-2.42882644	1.02E-13
HS3ST4	2.048937259	1.22E-18
HSPA1A	3.059663682	2.62E-21
HSPA1B	2.63453646	7.49E-17
HSPA6	4.789756785	3.03E-26
HTR1A	3.810910701	3.03E-26
HTR2B	2.826589998	1.82E-15
IFI44L	-2.000428647	1.98E-12
INHBA	3.050150611	1.22E-18
ITGB5	2.797474446	9.37E-20
KCNK9	2.243579859	5.13E-12
KIF26B	2.026416883	8.34E-13
KRT31	4.120775404	1.52E-16
KRT33B	3.500151694	7.06E-12
KRT34	4.016801799	2.53E-15
L1TD1	-1.943111035	1.62E-11
LGALS3	1.9403598	6.29E-12
LHX9	-2.101447191	1.04E-11
LIMCH1	1.872800611	1.41E-23
LIN28B	-2.865437872	9.06E-28
LPL	2.473995173	4.36E-21
MYL4	2.189925097	8.78E-12
NDRG1	2.002482897	5.40E-23
NDST4	-2.446380774	3.46E-19
NHS	-1.846459295	1.10E-19
NKX2-2	2.144896738	2.63E-11
NR4A2	1.959099345	8.01E-14
NXPH3	2.421847619	8.38E-13
OTOR	3.637188584	8.39E-17
PCDH11Y	2.10475491	1.49E-11
PLAG1	-1.794090145	1.89E-12
PTGDR2	2.128118729	1.10E-12
PTPRQ	-4.295245357	1.47E-20
RASL10A	3.855068264	1.07E-30
RGS6	2.422529019	1.04E-19
RHCG	1.932235479	9.03E-12

SCNN1G	2.124410007	2.84E-13
SEMA7A	1.925202754	5.50E-17
SERPINE1	2.320634042	1.09E-23
SH2D2A	1.931472479	2.52E-15
SHISA9	1.881593626	2.96E-12
SLC35F2	2.327910539	3.68E-19
SLCO1C1	3.035869614	6.57E-26
SOWAHB	1.960087246	2.52E-15
SPINK5	-3.144914762	9.09E-15
SV2C	-2.546574702	8.47E-16
SYT10	-2.253876006	3.09E-14
TAP1	1.955396556	1.69E-12
TMOD1	2.903499997	9.06E-28
TSHZ3	1.868857349	5.63E-12
TTLL2	2.705766923	1.66E-18

Negative Log2 fold values DECREASE in expression with age. They are more highly expressed at 9PCW.

Positive Log2 fold values INCREASE in expression with age. They are more highly expressed at 12 PCW.
