# Supplementary material for: HDBR Expression: A Unique Resource for Global and Individual Gene Expression Studies during Early Human Brain Development
Source: Front Neuroanat. 2016 Oct 26;10:86. doi: 10.3389/fnana.2016.00086 (PMC5080337; doi:10.3389/fnana.2016.00086)
Supplement: Supplementary file 4 [file Table4.pdf]

**Supplementary Table 4** **differential expression anterior-posterior cortex**

Genes that are differentially expressed between anterior and posterior cortex at **9 pcw**

| Gene Name | Log 2 fold | P adjusted |
|---|---|---|
| ZIC3 | 3.316820934 | 4.63E-21 |
| DCT | 3.209577832 | 5.32E-12 |
| GADL1 | 3.116578311 | 5.69E-16 |
| CYP26A1 | 3.033623659 | 3.75E-12 |
| MIR217HG | 2.586740936 | 4.78E-08 |
| LAMP5 | 2.494707745 | 2.16E-07 |
| MYBPC1 | 2.487607005 | 2.07E-07 |
| DCHS2 | 2.465400441 | 1.16E-11 |
| AE000661.37 | 2.291916082 | 6.61E-07 |
| LRRTM3 | 2.251127881 | 1.07E-07 |
| MOXD1 | 1.972086114 | 0.000234356 |
| TRDC | 1.926029601 | 0.000234356 |
| HKDC1 | 1.888456551 | 0.000520509 |
| ACTC1 | 1.887124989 | 0.00065774 |
| SPATA13 | 1.873128127 | 0.000218255 |
| ANKRD34B | 1.781708416 | 0.000714798 |
| GOLGA2P5 | 1.774802282 | 7.57E-11 |
| CTD-2140G10.2 | 1.773493427 | 0.000162022 |
| OTOG | 1.770753975 | 0.00286286 |
| KCNK9 | 1.715955148 | 2.28E-05 |
| PIP5K1B | 1.693719647 | 4.78E-08 |
| RORB | 1.691912163 | 3.28E-05 |
| CYP26B1 | 1.690272228 | 3.67E-05 |
| GIPR | 1.674604017 | 0.007743684 |
| LINC00643 | 1.6541064 | 0.003310335 |
| HILS1 | 1.641858318 | 0.009556352 |
| CNIH3 | 1.637172011 | 2.68E-05 |
| FAM196B | 1.63377138 | 0.008870445 |
| RP11-159K7.2 | 1.633666305 | 6.78E-05 |
| RLBP1 | 1.631551174 | 0.010110274 |
| CTB-12A17.2 | 1.617698181 | 2.16E-07 |
| RP11-844P9.2 | 1.615644149 | 0.000974515 |
| ARAP2 | 1.613256644 | 0.003659133 |
| HTR2A | 1.600195345 | 0.00028881 |
| NRK | 1.598215971 | 0.002140384 |

| | | |
|---|---|---|
| PTER | 1.590798504 | 8.82E-06 |
| SALL3 | 1.585766195 | 0.000187245 |
| CDH7 | 1.580273883 | 1.03E-08 |
| IL1RAPL2 | 1.565916233 | 0.017203942 |
| RP11-331K15.1 | 1.524803787 | 0.024192831 |
| SMCR2 | 1.519849323 | 0.026541759 |
| IMPA1P | 1.519264368 | 0.005962805 |
| TEC | 1.505161482 | 0.003384429 |
| RP1-137H15.2 | 1.503100791 | 0.028686765 |
| RCAN3 | 1.493077424 | 2.24E-07 |
| HHIP | 1.489927198 | 0.032019824 |
| USH1C | 1.464187669 | 0.007627456 |
| RP11-849I19.1 | 1.461171468 | 0.013848172 |
| LINC01285 | 1.45307862 | 0.022919544 |
| ETV1 | 1.444949634 | 4.28E-06 |
| ROBO1 | 1.433814992 | 1.45E-07 |
| CXXC4 | 1.418928923 | 0.008983871 |
| RP11-517P14.7 | 1.412157586 | 0.003450351 |
| SALL4 | 1.410132006 | 0.006568306 |
| LRRC9 | 1.409562211 | 0.008442412 |
| SOCS2 | 1.403587248 | 0.000520509 |
| IGSF11 | 1.402476565 | 0.000218255 |
| GRB14 | 1.400588027 | 0.007214822 |
| RIT2 | 1.394773327 | 0.014235703 |
| RP3-466P17.1 | 1.384298357 | 1.29E-06 |
| COL6A4P2 | 1.37120209 | 0.009556352 |
| PCDH17 | 1.36930736 | 0.018578223 |
| MYLK | 1.3647404 | 0.01015303 |
| WNT3 | 1.361005614 | 6.67E-05 |
| C11orf63 | 1.347683637 | 0.02891569 |
| SLC17A6 | 1.345440183 | 0.00457877 |
| NUS1P2 | 1.344425207 | 0.007727407 |
| CCDC175 | 1.344298448 | 0.03037099 |
| C18orf42 | 1.337492654 | 0.0466742 |
| POU3F2 | 1.328316267 | 0.010029015 |
| NTN4 | 1.323537562 | 0.000234356 |
| ANKRD29 | 1.317713457 | 6.15E-07 |
| EYS | 1.312153194 | 0.033268716 |
| FOLH1 | 1.292304635 | 1.18E-07 |
| RP11-384O8.1 | 1.291022683 | 0.035095866 |
| IGDCC4 | 1.290775688 | 1.72E-06 |
| RGAG1 | 1.288926273 | 0.000858828 |
| ZDHHC14 | 1.273409838 | 6.82E-06 |

| | | |
|---|---|---|
| HOPX | 1.268426033 | 0.036219784 |
| SYNJ2 | 1.243243967 | 0.005558463 |
| ME1 | 1.229041476 | 0.016128379 |
| VAT1L | 1.213489668 | 0.007327158 |
| CA8 | 1.20485907 | 0.016208501 |
| PHYH | 1.20342625 | 6.15E-07 |
| CCDC36 | 1.192401559 | 0.018773181 |
| SYNM | 1.189627814 | 0.02935281 |
| KITLG | 1.186059617 | 0.048343662 |
| SPAG1 | 1.165229985 | 0.019732216 |
| RP11-1109F11.3 | 1.164827062 | 0.007743684 |
| KCNV1 | 1.164327401 | 0.011400965 |
| PCDH8 | 1.164217503 | 0.036219784 |
| ST8SIA3 | 1.162692981 | 0.035977285 |
| ADCY1 | 1.161217488 | 0.000206635 |
| CRNDE | 1.161129637 | 0.00105812 |
| RHOJ | 1.153188308 | 0.00286286 |
| LINC01152 | 1.14973179 | 0.007214822 |
| TOX | 1.13750851 | 0.034350746 |
| NFIA-AS2 | 1.13592409 | 0.015618075 |
| NPY5R | 1.133032484 | 0.033268716 |
| RFTN2 | 1.126468384 | 0.034119921 |
| SP8 | 1.123387038 | 0.007743684 |
| RNF125 | 1.121703665 | 0.023689998 |
| RP3-428L16.2 | 1.095433337 | 0.005410852 |
| SOCS2-AS1 | 1.08176746 | 0.014311841 |
| BCAT1 | 1.067781707 | 0.036219784 |
| TENM2 | 1.0512605 | 0.008563057 |
| PDE1C | 1.045669936 | 2.49E-05 |
| AC068057.1 | 1.040251561 | 0.011908999 |
| AF186192.5 | 1.029889466 | 0.002269008 |
| FRK | 1.003643089 | 0.042375124 |
| NPTX1 | -1.02672212 | 0.034350746 |
| SYN2 | -1.03067815 | 0.03466247 |
| KIAA1324 | -1.08399978 | 0.005378528 |
| FZD7 | -1.09013391 | 1.87E-07 |
| ATOH7 | -1.09187289 | 0.013848172 |
| CD27-AS1 | -1.12265269 | 4.43E-05 |
| PTPN3 | -1.18490074 | 0.002393295 |
| PPARG | -1.2448199 | 0.000351408 |
| DPP10-AS1 | -1.2532731 | 0.01586224 |
| CNGA3 | -1.26976345 | 3.28E-05 |
| DIRAS3 | -1.27249822 | 0.006195153 |
| FBLN7 | -1.31511014 | 0.00105812 |
| MICAL2 | -1.3646685 | 0.033558184 |
| SHISA6 | -1.38131765 | 2.19E-09 |

| | | |
|---|---|---|
| RP11-469H8.6 | -1.44220555 | 0.013848172 |
| HRK | -1.45189445 | 0.035095866 |
| RP11-215G15.5 | -1.46059807 | 0.003659133 |
| DCLK3 | -1.46752486 | 1.32E-07 |
| ECEL1 | -1.47979885 | 0.036726237 |
| MKX | -1.48089544 | 0.028478615 |
| CHST15 | -1.50175486 | 0.000120101 |
| SPINK5 | -1.50997083 | 0.00105812 |
| TAC3 | -1.51108222 | 0.021722228 |
| CHRNA3 | -1.53933121 | 8.82E-06 |
| NECAB1 | -1.59234887 | 0.015618075 |
| AC004862.6 | -1.61843195 | 0.002843333 |
| PDPN | -1.67599432 | 3.25E-09 |
| CHODL | -1.68190687 | 7.21E-06 |
| RP1-212P9.2 | -1.82140837 | 0.000267376 |
| DGKK | -2.02929528 | 0.000234356 |
| NR2F1 | -2.38816284 | 3.77E-07 |
| CDH9 | -2.41305581 | 6.15E-07 |
| NR2F1-AS1 | -2.43072095 | 5.60E-09 |
| OCA2 | -2.68624169 | 1.08E-20 |
| MAS1 | -3.11502747 | 9.56E-24 |
| FGFR3 | -4.82759528 | 1.05E-39 |

Positive Log2 fold value is higher in the anterior cortex.

Negative Log2 fold value is higher in the posterior cortex.

Genes that are differentially expressed between anterior and posterior cortex at **12 pcw**

| Gene Name | Log 2 fold | P adjusted |
|---|---|---|
| SLC17A8 | -1.98648884 | 2.84E-05 |
| PDZRN3 | -1.80670657 | 4.56E-05 |
| GIPR | -1.79900599 | 0.00020008 |
| RGS8 | -1.79878044 | 5.74E-05 |
| WIF1 | -1.7572201 | 4.56E-05 |
| ST8SIA5 | -1.7517511 | 5.74E-05 |
| CA8 | -1.74483807 | 0.000125193 |
| SYNDIG1L | -1.70635595 | 0.000382599 |
| TLL1 | -1.70567259 | 0.000404269 |
| ADAM33 | -1.66330276 | 0.000382599 |
| GRIN2C | -1.64669938 | 0.000404269 |
| MYBPHL | -1.63505047 | 0.000382599 |

| | | |
|---|---|---|
| GABRQ | -1.61856872 | 0.000302197 |
| GRM4 | -1.61773437 | 4.56E-05 |
| BRINP2 | -1.56031888 | 0.000733991 |
| CNTN6 | -1.55483391 | 0.000738993 |
| RP11-742D12.2 | -1.53757292 | 0.001974768 |
| ZBTB7C | -1.52650847 | 0.000757096 |
| NPY5R | -1.52610876 | 0.001127388 |
| KCNMB2 | -1.51266405 | 0.000302197 |
| LAMP5 | -1.51096481 | 0.002313725 |
| KRT19 | -1.49972027 | 0.001154186 |
| RP4-555D20.2 | -1.49923158 | 0.001081937 |
| RP11-13K12.1 | -1.4985507 | 0.002148024 |
| COL12A1 | -1.49485988 | 4.56E-05 |
| SLIT3 | -1.46399933 | 0.002148024 |
| LRFN2 | -1.45427915 | 0.001550305 |
| THRB | -1.44769574 | 0.00093725 |
| UNC5C | -1.4459131 | 0.004359524 |
| ATP5F1P5 | -1.43900707 | 0.004667426 |
| SERTM1 | -1.43867462 | 0.000302197 |
| ERBB4 | -1.40520211 | 0.002272964 |
| GPR1 | -1.40425059 | 0.00388003 |
| SCGN | -1.40044333 | 0.003990401 |
| SLC25A45 | -1.39577583 | 0.004667426 |
| DLX6-AS1 | -1.38833688 | 0.007336626 |
| DPF3 | -1.38487038 | 0.001064948 |
| NAALAD2 | -1.38283179 | 0.001154186 |
| SPON1 | -1.37641615 | 0.008428975 |
| NPAS1 | -1.37235405 | 0.00093725 |
| PTGS2 | -1.37180351 | 0.003395648 |
| PIP5K1B | -1.36515377 | 0.001321734 |
| ARAP2 | -1.3617944 | 0.001465728 |
| EYA1 | -1.3599742 | 0.001664042 |
| NRIP3 | -1.35825578 | 0.007162924 |
| SYNJ2 | -1.35516512 | 0.00043179 |
| ABI3BP | -1.35207785 | 0.006096373 |
| RCAN2 | -1.35064863 | 0.001013927 |
| KCNC2 | -1.34936804 | 0.008101362 |
| KCNIP4 | -1.34702996 | 0.004359524 |
| CLDN1 | -1.34565978 | 0.008901471 |
| MDGA1 | -1.34347531 | 6.22E-06 |
| ADAMTS14 | -1.34098624 | 0.003656903 |
| ABCA1 | -1.33980358 | 0.001038083 |
| ZNF385A | -1.33852827 | 0.00093725 |
| COLEC12 | -1.33752322 | 0.001974768 |
| KIRREL3 | -1.32776612 | 5.74E-05 |
| RARB | -1.32568269 | 0.012219823 |

| | | |
|---|---|---|
| C11orf63 | -1.32219587 | 0.005681219 |
| BEST4 | -1.31798197 | 0.004359524 |
| CDCP1 | -1.31387854 | 0.012993273 |
| ANXA2P2 | -1.31147607 | 0.01085732 |
| TRIM71 | -1.30893981 | 0.000652415 |
| CPNE8 | -1.30510529 | 0.012361349 |
| MGAT4C | 1.301265076 | 0.000733991 |
| CARD10 | -1.29598947 | 0.001570893 |
| NRXN3 | -1.29295984 | 0.005648568 |
| ITPR2 | -1.28973389 | 0.000685751 |
| RPH3A | -1.28528832 | 0.007167369 |
| KLHL4 | -1.2778529 | 0.000652415 |
| PRLHR | -1.27775997 | 0.00595939 |
| TUNAR | -1.27771921 | 0.012391233 |
| GPNMB | -1.27708249 | 0.018339787 |
| RP11-796G6.1 | -1.27544126 | 0.01779328 |
| TAC1 | -1.26688581 | 0.018339787 |
| CAMK1G | -1.26533311 | 0.008901471 |
| LGI4 | -1.26441216 | 0.003789042 |
| ALCAM | -1.2636627 | 0.000154585 |
| RP11-679B19.1 | -1.26078352 | 0.00093725 |
| SYTL5 | -1.25964198 | 0.014099385 |
| NPY1R | -1.25527984 | 0.002743176 |
| PLCXD3 | -1.24942163 | 0.005408338 |
| VWA5B1 | -1.24878462 | 0.008101362 |
| GAD1 | -1.24767674 | 0.014099385 |
| UST | -1.23692034 | 0.007187283 |
| SLC32A1 | -1.23381733 | 0.02207139 |
| GHR | -1.23335349 | 0.00041953 |
| YPEL2 | -1.23101397 | 0.005648568 |
| AE000661.37 | -1.22904233 | 0.025233298 |
| GRM7 | -1.22778605 | 0.022323189 |
| TBX21 | -1.22760534 | 0.016841981 |
| DLX1 | -1.22638145 | 0.023500504 |
| BCYRN1 | -1.21380067 | 0.018339787 |
| ADARB2 | -1.21282483 | 0.018683871 |
| FBLN5 | -1.21164866 | 0.028460906 |
| PTPRR | -1.20580984 | 0.000712745 |
| CNIH3 | -1.20429308 | 0.019277258 |
| CBLN2 | -1.19541634 | 0.024641462 |
| GRIP2 | -1.19485505 | 4.09E-05 |
| TGFB2 | -1.19266424 | 0.025029895 |
| ZCCHC12 | -1.19165591 | 0.012440962 |
| PTPRT | -1.19154735 | 0.01147044 |
| PLXNB3 | -1.18917096 | 0.01640914 |

| | | |
|---|---|---|
| RP11-449L23.2 | -1.18453611 | 0.031722112 |
| GABRA1 | -1.18390177 | 0.028544847 |
| DNAH10 | -1.18059073 | 0.025182785 |
| CSMD3 | -1.17754633 | 0.019668238 |
| PRRT4 | -1.17716954 | 0.00954253 |
| RGS11 | -1.17062457 | 0.000733991 |
| PART1 | -1.16599705 | 0.03819006 |
| LINC00277 | -1.16528293 | 0.013293208 |
| CACNA1D | -1.16213074 | 0.000644946 |
| DLX5 | -1.16139437 | 0.035536552 |
| FAM222A | -1.1595874 | 0.030011208 |
| BCL6 | -1.15956033 | 0.003656903 |
| CDH6 | -1.15065139 | 0.010297013 |
| SYNM | -1.14944412 | 0.003112694 |
| GPR17 | -1.14274411 | 0.031733934 |
| COL15A1 | -1.13962118 | 0.004667426 |
| CYP26A1 | -1.1368572 | 0.042761997 |
| RP11-108M9.3 | -1.13585333 | 0.039254064 |
| ANGPT1 | -1.12497518 | 0.025828658 |
| LGR4 | -1.12456371 | 0.030426116 |
| TC2N | -1.11646345 | 0.036424633 |
| FGF1 | -1.11483867 | 0.041315732 |
| FREM1 | -1.10454394 | 0.030271439 |
| CYGB | -1.10252841 | 0.029063441 |
| NTN1 | -1.09853822 | 0.032962239 |
| LMO4 | -1.09495078 | 0.023900177 |
| RP11-272L13.3 | -1.09230503 | 0.019496855 |
| STK32B | -1.09208272 | 0.043585879 |
| GREB1 | -1.09045041 | 0.048499065 |
| MIR137HG | -1.08680114 | 0.01779328 |
| HR | -1.08624737 | 0.030639231 |
| CHRM2 | -1.08607741 | 0.030503459 |
| LINC01305 | -1.07963752 | 0.046526097 |
| PLA2R1 | -1.07685542 | 0.035536552 |
| RP11-517P14.7 | -1.07427896 | 0.013835774 |
| ABCA12 | -1.07110173 | 0.038121398 |
| RP11-351J23.2 | -1.06748398 | 0.030271439 |
| LRRTM3 | -1.06156755 | 0.017627886 |
| ZMAT4 | -1.05985104 | 0.017611929 |
| HCRTR2 | -1.05763347 | 0.030579987 |
| PLS3 | -1.0563492 | 0.013468674 |
| PCDH19 | -1.05514921 | 0.035536552 |
| RP11-9G1.3 | -1.04989341 | 0.010345895 |
| FZD4 | -1.04489298 | 0.00509972 |

| | | |
|---|---|---|
| SYN3 | -1.0447323 | 0.027018371 |
| COLGALT2 | -1.04447481 | 0.038121398 |
| KLHL29 | -1.04130448 | 0.012901845 |
| IFIT3 | -1.03847394 | 0.034889917 |
| ITPR3 | -1.03785464 | 0.013293208 |
| LRRC9 | -1.036517 | 0.00746993 |
| MLIP | -1.03581536 | 0.000605117 |
| RP11-713C5.1 | -1.03488682 | 0.047739216 |
| TMEFF2 | -1.0346771 | 0.031193902 |
| LYPD6B | -1.03165112 | 0.045362743 |
| ZNF114 | -1.02967851 | 0.007675266 |
| MIR770 | -1.02506056 | 0.031733934 |
| KRT13 | -1.02361414 | 0.048048637 |
| ANXA2 | -1.02313622 | 0.002587916 |
| TCEA3 | -1.0227556 | 0.042761997 |
| RP11-490G2.2 | -1.02009536 | 0.042761997 |
| PPP2R1B | -1.01433257 | 0.012391233 |
| C2orf72 | -1.01021113 | 0.04938823 |
| OLIG2 | -1.0070065 | 0.038507579 |
| RASGRF2 | -1.00539728 | 0.012325442 |
| ETS2 | -1.00130727 | 0.000520869 |
| PTGES3P2 | 1.002540735 | 0.000382599 |
| PAPPA2 | 1.009824411 | 0.030627497 |
| CHRNA3 | 1.015743977 | 0.017991722 |
| RPL12L3 | 1.027108261 | 0.036862095 |
| CTD-2620I22.7 | 1.076004754 | 0.035536552 |
| SLC7A10 | 1.094301561 | 0.044338822 |
| RP11-932O9.10 | 1.096634206 | 0.031132905 |
| CYP26B1 | 1.120025742 | 0.019668238 |
| SNORD15B | 1.122684366 | 0.043099727 |
| COX7A1 | 1.133388921 | 0.005680874 |
| KIF25-AS1 | 1.14935424 | 0.026428148 |
| MDFI | 1.167748363 | 0.004113725 |
| RPS12P26 | 1.263538249 | 0.008882413 |
| LLNLF-158E9.1 | 1.271713817 | 0.003990401 |
| RP5-1024C24.1 | 1.3538704 | 0.000600792 |
| CARTPT | 1.365887388 | 0.001664042 |
| RP11-677M24.1 | 1.418327114 | 0.002952903 |

Positive Log2 fold value is higher in the anterior cortex.

Negative Log2 fold value is higher in the posterior cortex.

Genes that are differentially expressed between anterior and posterior cortex at both 9 and 12 pcw

| Gene Name | Gene Type | 9 PCW | | 12 PCW | |
|---|---|---|---|---|---|
| | | Log 2 fold | P adjusted | Log 2 fold | P adjusted |
| CYP26A1 | Protein coding | 3.03 | 3.75E-12 | 1.14 | 0.042762 |
| LAMP5 | Protein coding | 2.49 | 2.16E-07 | 1.51 | 0.002314 |
| AE000661.37 | Processed transcript | 2.29 | 6.61E-07 | 1.23 | 0.025233 |
| LRRTM3 | Protein coding | 2.25 | 1.07E-07 | 1.06 | 0.017628 |
| PIP5K1B | Protein coding | 1.69 | 4.78E-08 | 1.37 | 0.001322 |
| CYP26B1 | Protein coding | 1.69 | 3.67E-05 | 1.12 | 0.019668 |
| GIPR | Protein coding | 1.67 | 0.007744 | 1.8 | 0.0002 |
| CNIH3 | Protein coding | 1.64 | 2.68E-05 | 1.2 | 0.019277 |
| ARAP2 | Protein coding | 1.61 | 0.003659 | 1.36 | 0.001466 |
| RP11-517P14.7 | Unprocessed pseudogene | 1.41 | 0.00345 | 1.07 | 0.013836 |
| LRRC9 | Protein coding | 1.41 | 0.008442 | 1.04 | 0.00747 |
| C11orf63 | Protein coding | 1.35 | 0.028916 | 1.32 | 0.005681 |
| SYNJ2 | Protein coding | 1.24 | 0.005558 | 1.36 | 0.000432 |
| CA8 | Protein coding | 1.2 | 0.016209 | 1.75 | 0.000125 |
| SYNM | Protein coding | 1.19 | 0.029353 | 1.15 | 0.003113 |
| NPY5R | Protein coding | 1.13 | 0.033269 | 1.53 | 0.001127 |
| CHRNA3 | Protein coding | 1.54 | 8.82E-06 | 1.02 | 0.017992 |

**Red** Log2 fold values are higher in anterior

**Blue** log2 fold values are higher in posterior
